# Primary tumour location, molecular alterations, treatments, and outcome in a population-based metastatic colorectal cancer cohort

**DOI:** 10.1038/s44276-025-00156-z

**Published:** 2025-05-28

**Authors:** Emerik Osterlund, Klara Hammarström, Luís Nunes, Lucy Mathot, Artur Mezheyeuski, Tobias Sjöblom, Bengt Glimelius

**Affiliations:** 1 Department of Immunology, Genetics and Pathology, Uppsala University, Uppsala, Sweden; 2 Department of Molecular Oncology, Institute for Cancer Research, Oslo University Hospital, Oslo, Norway; 3 Molecular Oncology Group, Vall d’Hebron Institute of Oncology, Barcelona, Spain

## Abstract

**Background:**

Metastatic colorectal cancer (mCRC) patients in trials are selected. The aim was to study mCRC features population-based.

**Methods:**

All 765 mCRC patients in the Uppsala region, Sweden, 2010–2020 were identified and analysed for RAS (*n* = 356/708) and *BRAF*-V600E (*n* = 123/708) mutations (mt) and deficient mismatch repair (dMMR, *n* = 58/643).

**Results:**

Right colon primary tumours were associated with *BRAF*-V600Emt and dMMR and had worse median overall survival (mOS) than left colon or rectal mCRC. RAS&*BRAF* wildtype (wt) and proficient MMR were seen in 22%, 45%, and 31% of right colon, left colon, and rectum, respectively. Patients with right colon primaries received best supportive care only more often (34% vs 25% vs 24%) and metastasectomy less often (21% vs 31% vs 33%) than left colon and rectal primaries. In molecularly homogeneous subgroups (RAS&*BRAF*wt/RASmt/*BRAF*-V600Emt/dMMR) no difference in mOS were seen between right and left colon primaries, whereas rectal primaries had better mOS (26/15/8/9 vs 24/21/8/8 vs 32/23/6/NA months, respectively). This was also the case in homogenous treatment groups. Primary tumour location turned non-significant in multivariable OS analyses.

**Conclusions:**

The high variation of *BRAF*-V600Emt, RASmt, dMMR, and treatment allocation population-based per primary tumour location explain the poor outcome in right-sided cancers.

## Introduction

Colorectal cancer (CRC) is the third most common cancer and the second leading cause for cancer death worldwide [1]. At diagnosis, distant metastases are found in about 25% of the patients and up to 20% will recur after curative surgery. Medical treatment for metastatic CRC (mCRC) has advanced substantially [2], resulting in marked survival prolongation, with recent clinical trials reporting a median overall survival (mOS) exceeding 30 months [2, 3]. Surgery for metastatic disease has also contributed to the improved outcome [2, 4].

This marked progress has not been reported in population-based patient series. Rather, mOS in the order of 12–15 months is reported [5–10]. Patients in clinical trials or treated at dedicated hospitals are younger, have better Eastern Cooperative Oncology Group performance status (ECOG-PS), fewer comorbidities, and underrepresentation of certain metastatic sites. Patients included in recent clinical trials have been molecularly selected and subgroups with poor prognosis, such as RAS mutations (mt), *BRAF*-V600Emt, and deficient mismatch repair (dMMR), are more frequent population-based than in trials/hospital-based series [11–13].

For decades, patients with mCRC from different parts of the colon and rectum were handled similarly since they had the same prognosis and responded similarly to chemotherapy and besides colon and rectum, primary tumour location was seldom reported [14]. After the introduction of biologic agents, studies have reported a worse prognosis in patients with right colon tumours indicating that they should be treated differently [2, 15, 16]. The latter particularly relates to the use of epidermal growth factor receptor (EGFR)-inhibitors, and recommendations not to use these in right-sided tumours have emerged [2, 17–19]. Studies have also suggested that the colorectum should be divided beyond sidedness [20–23].

To better understand the relevance of different prognostic factors, including primary tumour location, all patients with mCRC in a defined population were identified and clinicopathological information collected. Diagnostic tumour material was, whenever sufficient, analysed for RAS, *BRAF*, and *PIK3CA* mutations, and MMR-status. The aim was to describe in detail clinical characteristics, treatments used, and outcomes in a population-based mCRC cohort, where selection has been reduced to a minimum, with focus on the relevance of primary tumour location and molecular alterations.

## Methods

### Patients and clinicopathological data collection

All patients living in the Uppsala region (population 388,000 in 2020) diagnosed with a primary CRC between January 2010 and December 2020 have been prospectively registered in the Swedish Colorectal Cancer Registry (coverage virtually 100%) [24] where relevant clinical information is documented. Biomaterials (blood and tissue) were collected in the Uppsala-Umeå Comprehensive Cancer Consortium [25]. The clinical records at Akademiska sjukhuset, were searched annually for missed recurrences, and all diagnoses of synchronous disease re-evaluated [24].

All treatments and care activities were performed according to guidelines. Systemic therapy (treatment intent either palliative, neoadjuvant, conversion, or adjuvant after metastasectomy) was given according to the Swedish national cancer care programme, which closely adheres to the European Society for Medical Oncology (ESMO)-guidelines [2, 3, 26]. EGFR-inhibitors were not given if the tumour was RASmt or *BRAF*-V600Emt [2, 27, 28]. During the inclusion period, sidedness was generally not considered in the decision of whether to give EGFR-inhibitors.

### Molecular analyses

Tumour tissue from biopsies and/or surgical specimens was collected from primary tumours and/or metastases. Molecular analyses were done in routine healthcare for *KRAS* (codons 12, 13, and 61) and *BRAF*-V600E mutations using pyrosequencing [29] prior to November 2014 (16 patients were tested only for *KRAS-* and *BRAF*-V600E mutations; they were included in the RAS&*BRAF* wildtype [wt] group). Since December 2014, next-generation sequencing (NGS) panels have been used to analyse hotspot mutations in *KRAS* and *NRAS* (codons 12, 13, 61, and 146), *BRAF*-V600E, and since autumn 2016 *PIK3CA*. MMR-status was analysed in clinical routine for all patients using immunohistochemistry (IHC) since 2020, and as targeted testing before this.

Diagnostic slides were re-examined to identify material for additional molecular testing for as many cases as possible. Whenever fresh frozen tumour material with ≥20% tumour cell content and matched blood or normal tissue were available, whole-genome sequencing (WGS) was performed [30]. The coding regions of the *KRAS* and *NRAS* genes, *BRAF*-V600E, and *PIK3CA* were considered here.

In the cases without frozen material, formalin-fixed paraffin-embedded (FFPE) material was used for DNA extraction, if tumour content ≥10%. It was not allowed to consume all tumour material because of potential clinical use. DNA was extracted from FFPE, using the NGEx^®^ FFPE DNA purification kit (Oncodia, Sweden) and sequenced with Ion Torrent^TM^ Oncomine^TM^ Tumour Mutation Load assay (Thermo-Fisher Scientific, MA, USA) covering 1.7 Mb across 409 cancer-driver genes, allowing for tumour mutation burden (TMB) assessment [31]. The coding regions of the *KRAS* and *NRAS* genes, *BRAF*-V600E, and *PIK3CA* were considered here. Genetic testing was part of clinical routine in 603 samples, using NGS in 419 (77 not analysed in clinical routine), and using WGS in 187 (10 not analysed in clinical routine or using NGS). Samples of 5 patients had no invasive tumour detected and for 52 patients the material was too scarce.

Microsatellite instability (MSI) was assessed with the TrueMark^TM^ MSI Assay kit (Thermo-Fisher Scientific, MA, USA) as a multiplex PCR with subsequent fragment analysis. If ≥4/13 markers were unstable the tumour was classified as MSI-high (denoted dMMR). WGS was used for determining MSI-status using MSIsensor2 tumour-normal paired mode [30, 32]. dMMR was assessed using IHC for MSH6, PMS2, MLH1, and MSH2 [12], with limited material only MSH6 and PMS2 were stained. For 155 patients, TMB > 10 by the Ion Torrent^TM^ Oncomine^TM^ Tumour Mutation Load assay was used as a proxy for dMMR [33].

### Statistical analyses

The STROBE-statement for cohort studies was adhered to [34].

Categorical data were presented as proportions, with percentages, and Chi-square or Fischer’s exact test used for comparisons. Mann–Whitney or Kruskal–Wallis’s tests were used for continuous variables. Two-tailed *p*-values < 0.05 were considered statistically significant.

OS was estimated from mCRC diagnosis to death or censored if alive at last follow-up (data cut-off 16 March 2023) using Kaplan–Meier. OS comparisons were performed using Cox regression models with 95% confidence intervals (95% CI). A multivariable Cox regression model was constructed where statistically significant and clinically relevant variables were included. Conditional landmark analysis for OS was used for assessing whether guarantee time bias influenced the effect of treatments. Proportional hazard assumption was checked visually using Schoenfeld residuals, no clear violations were seen. All statistical analyses were done using SPSS statistical software versions 27 and 29 (IBM Corporation, Armonk, NY, USA).

## Results

### Characteristics of the cohort

Between January 2010 and December 2020, 2,114 patients living in the Uppsala region were diagnosed with CRC. Of these, 510 (24%) had synchronous metastases and 255 (12%) developed metachronous metastases before March 2023. Totally, 765 mCRC patients constitute the final cohort.

Primary tumour location was right colon (caecum [including appendix] to transverse colon) in 291 (38%), left colon (splenic flexure to sigmoid colon) in 207 (27%), and rectum (distal 15 cm) in 262 (34%). Characteristics according to primary tumour location are shown in Table 1. Median age was higher for colon cancers (right colon 74 years, left colon 72 years) than for rectal cancers (70 years, *p* = 0.001). Right colon tumours were more often seen in females and their ECOG-PS at diagnosis of metastatic disease was worse. Synchronous disease was more common in left colon tumours than in right colon and rectal tumours. Liver and lung metastases were less common in right colon tumours than in left colon and rectal tumours, whereas peritoneal metastases were most frequent in right colon tumours.

**Table 1:** Patient characteristics according to primary tumour location.

		Right colon		Left colon		Rectum		Total^a^		*p*-value
		291	38%	207	27%	262	34%	765	100%	
Median age (range)		74 (32–96)		72 (28–92)		70 (35–99)		72 (28–99)		0.001
Total		291	100%	207	100%	262	100%	765	100%	
Age groups	≤70 years	104	36%	85	41%	122	47%	313	41%	0.035
	>70 years	187	64%	122	59%	140	53%	452	59%	
Sex	Male	132	45%	114	55%	156	60%	405	53%	0.003
	Female	159	55%	93	45%	106	41%	360	47%	
Primary resection	No	99	34%	93	45%	143	55%	339	44%	<0.001
	Yes	192	66%	114	55%	119	45%	426	56%	
Tumour grade	Low	152	55%	138	74%	175	80%	467	68%	<0.001
	High	122	45%	49	26%	44	20%	216	32%	
	Missing	17	–	20	–	43	–	82	–	–
Presentation of metastases	Synchronous	187	64%	153	74%	166	63%	510	67%	0.031
	Metachronous	104	36%	54	26%	96	37%	255	33%	
Number of metastatic sites	1	143	49%	91	44%	129	49%	367	48%	0.663
	2	104	36%	76	37%	92	35%	273	36%	
	3–5	44	15%	40	19%	41	16%	125	16%	
Metastatic sites	Liver	176	61%	149	72%	170	65%	498	65%	0.029
	Lung	86	30%	81	39%	138	53%	305	40%	<0.001
	Peritoneum	115	40%	59	29%	30	12%	206	27%	<0.001
	Lymph nodes	82	28%	42	20%	71	27%	196	26%	0.112
	Bone	9	3%	9	4%	12	5%	30	4%	0.630
	Brain	5	2%	5	2%	6	2%	16	2%	0.839
	Other	29	10%	28	14%	23	9%	80	10%	0.232
ECOG PS	0	75	26%	70	34%	105	40%	252	33%	0.003
	1	102	35%	58	28%	83	32%	243	32%	
	2–4	114	39%	78	38%	74	28%	269	35%	
	Missing	–	–	1	–	–	–	1	–	–
Mutation status	*BRAF*-V600Emt	89	34%	21	11%	12	5%	123	17%	<0.001
	RASmt	117	45%	88	45%	158	64%	365	52%	
	RAS&*BRAF*wt	57	22%	86	44%	77	31%	220	31%	
	Not tested	28	–	12	–	15	–	57	–	–
MMR-status	pMMR	150	79%	130	96%	157	98%	438	90%	<0.001
	dMMR	41	21%	6	4%	3	2%	50	10%	
	Not tested	100	–	71	–	102	–	277	–	–
Type of treatment	Metastasectomy	62	21%	64	31%	87	33%	213	28%	0.006
	Systemic therapy only	129	44%	90	44%	113	43%	334	44%	
	Best supportive care	100	34%	52	25%	62	24%	217	28%	
	Not known	–	–	1	–	–	–	1	–	–

*dMMR* deficient mismatch repair, *ECOG PS* Eastern Cooperative Oncology Group performance status, *MMR* mismatch repair, *pMMR* proficient mismatch repair

^a^5 with unknown/multiple primary tumours not presented separately.

### Molecular characteristics of the tumours according to tumour site

All patients had a morphologically verified adenocarcinoma. Tumour grade was possible to characterise in 683 (89%) patients. High-grade tumours were more frequent in right colon than in left colon and rectum (Table 1), and in *BRAF*-V600Emt compared with RASmt and RAS&*BRAF*wt (Table 2).

**Table 2:** Patient characteristics according to mutation status.

		*BRAF*-V600Emt		RASmt		RAS&*BRAF*wt		Total^a^		*p*-value
		123	17%	365	52%	220	31%	765	100%	
Median age (range)		73 (36–96)		72 (30–99)		70 (28–95)		72 (28–99)		0.078
Total		123	100%	365	100%	220	100%	765	100%	
Age groups	≤70 years	45	37%	150	41%	107	49%	313	41%	0.066
	>70 years	78	63%	215	59%	113	51%	452	59%	
Sex	Male	49	40%	198	54%	128	58%	405	53%	0.004
	Female	74	60%	167	46%	92	42%	360	47%	
Primary tumour	Right colon	89	73%	117	32%	57	26%	291	38%	<0.001
location	Left colon	21	17%	88	24%	86	39%	207	27%	
	Rectum	12	10%	158	44%	77	35%	262	34%	
	Unknown/multiple	1	–	2	–	–	–	5	–	–
Primary resection	No	57	46%	154	42%	81	37%	339	44%	0.199
	Yes	66	54%	211	58%	139	63%	426	56%	
Tumour grade	Low	45	38%	253	79%	147	72%	467	68%	<0.001
	High	73	62%	68	21%	57	28%	216	32%	
	Missing	5	–	44	–	16	–	82	–	–
Presentation of metastases	Synchronous	81	66%	227	62%	152	69%	510	67%	0.232
	Metachronous	42	34%	138	38%	68	31%	255	33%	
Number of metastatic sites	1	53	43%	173	47%	112	51%	367	48%	0.223
	2	53	43%	123	34%	76	35%	273	36%	
	3–5	17	14%	69	19%	32	15%	125	16%	
Metastatic sites	Liver	63	51%	246	67%	147	67%	498	65%	0.003
	Lung	39	32%	183	50%	65	30%	305	40%	<0.001
	Peritoneum	49	40%	79	22%	68	31%	206	27%	<0.001
	Lymph nodes	41	33%	86	24%	50	23%	196	26%	0.062
	Bone	6	5%	16	4%	7	3%	30	4%	0.692
	Brain	5	4%	8	2%	1	1%	16	2%	0.056
	Other	15	12%	36	10%	27	12%	80	10%	0.599
ECOG PS	0	25	20%	144	40%	81	37%	252	33%	<0.001
	1	36	29%	112	31%	74	34%	243	32%	
	2–4	62	50%	109	30%	65	30%	269	35%	
	Not available	–	–	–	–	–	–	1	–	–
MMR-status	pMMR	56	67%	235	96%	139	91%	438	90%	<0.001
	dMMR	28	33%	9	4%	13	9%	50	10%	
	Not tested	39	–	121	–	66	–	277	–	–
Type of treatment	Metastasectomy	18	15%	113	31%	79	36%	213	28%	<0.001
	Systemic therapy only	62	50%	169	46%	93	42%	334	44%	
	Best supportive care	43	35%	83	23%	48	22%	217	28%	
	Not known	–	–	–	–	–	–	1	–	–

*dMMR* deficient mismatch repair, *ECOG PS* Eastern Cooperative Oncology Group performance status, *MMR* mismatch repair, *pMMR* proficient mismatch repair

^a^57 non-analysed tumours not presented separately

Molecular analyses were performed for 708 (93%) cases, with similar frequencies for each primary tumour location. Not molecularly tested patients were older, had less primary tumour resections, synchronous presentation more often, poorer ECOG-PS, and received active treatment less often (Supplementary Table S1). Of the 708 tested patients, 123 (17%) had *BRAF*-V600Emt; being more common in right colon compared with left colon and rectum (Table 1). RASmt was seen in 363 (52%) tumours and was less common in right colon and left colon compared with rectum. *PIK3CA*mt was seen in 62/248 (25%) of right colon, in 35/185 (19%) of left colon, and in 29/235 (12%) of rectal tumours (*p* = 0.002).

Patient characteristics for each mutation group are shown in Table 2. No differences in age, presentation of metastases, or number of metastatic sites were seen. Female sex was more common and ECOG-PS worse among *BRAF*-V600Emt compared with RASmt and RAS&*BRAF*wt. Liver metastases were less common and peritoneal metastases more common in *BRAF*-V600Emt compared with RASmt and RAS&*BRAF*wt. Lung metastases were more common in RASmt compared with *BRAF*-V600Emt and RAS&*BRAF*wt.

**Fig. 1:**
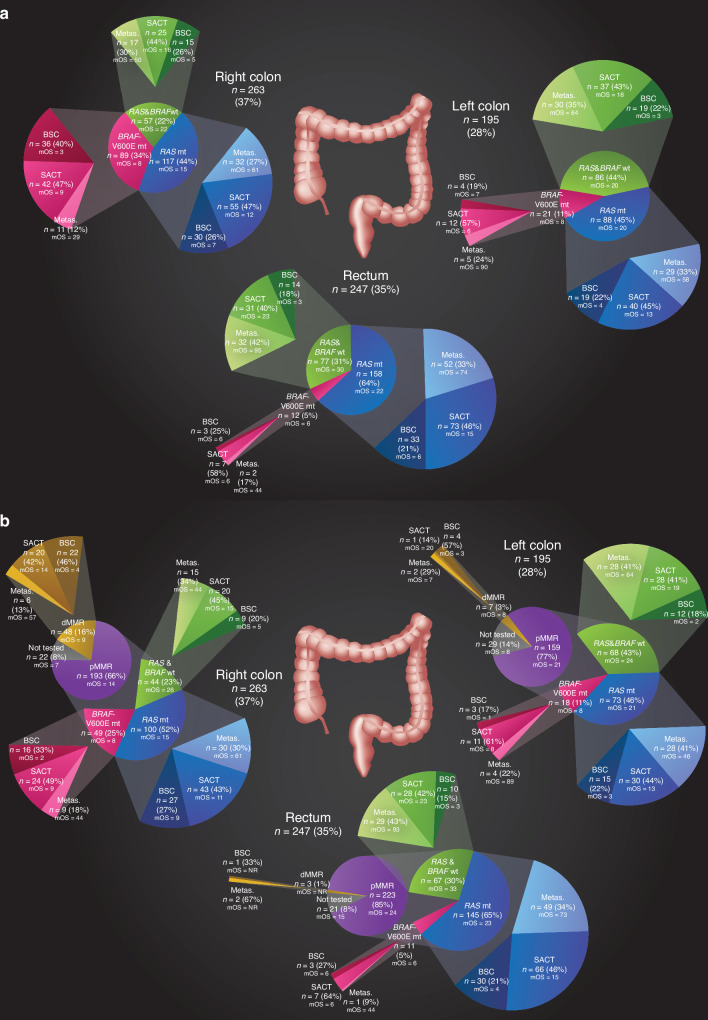
Primary tumour location divided by molecular alterations and treatment groups, and median overall survival for each respective subgroup. According to mutation status (**a**), and further separated by mismatch repair status (**b**).

MMR-status was assessed by IHC, PCR, or WGS in 488 or by TMB as a proxy in 155 tumours. Fifty-eight (9%) of the 643 (84%) tumours characterised for MMR had dMMR. dMMR was more common in right colon than left colon and rectum (Table 1, Fig. 1B), and in *BRAF*-V600Emt compared with RASmt and RAS&*BRAF*wt (Table 2). Characteristics according to MMR-status are presented in Table 3. dMMR patients were older, more often female, had worse ECOG-PS, and had high-grade tumours more often compared with pMMR. No difference in presentation of metastases or number of metastatic sites were seen. Peritoneal metastases were more common in dMMR compared with pMMR, whereas no differences were seen for other metastatic sites. Patients not tested for MMR were more similar to patients with pMMR. Results were in line if patients only analysed with TMB as a proxy for MMR were excluded (data not shown).

**Table 3:** Patient characteristics according to mismatch repair status.

		pMMR		dMMR		Not tested		Total		*p*-value*
		585	76%	58	8%	122	16%	765	100%	
Median age (range)		71 (30–99)		74 (36–95)		75 (28–96)		72 (28–99)		0.012
Total		585	100%	58	100%	122	100%	765	100%	
Age groups	≤70 years	258	44%	18	31%	37	30%	313	41%	0.055
	>70 years	327	56%	40	69%	85	70%	452	59%	
Sex	Male	321	55%	16	28%	68	56%	405	53%	<0.001
	Female	264	45%	42	72%	54	44%	360	47%	
Primary tumour	Right colon	199	34%	48	83%	44	37%	291	38%	<0.001
location	Left colon	159	27%	7	12%	41	34%	207	27%	
	Rectum	225	39%	3	5%	34	29%	262	34%	
	Unknown/multiple	2	–	–	–	3	–	5	–	–
Primary resection	No	232	40%	14	24%	93	76%	339	44%	0.02
	Yes	353	60%	44	76%	29	24%	426	56%	
Tumour grade	Low	396	74%	14	25%	57	63%	467	68%	<0.001
	High	140	26%	43	75%	33	37%	216	32%	
	Missing	49	–	–	–	32	–	82	–	–
Presentation of metastases	Synchronous	380	65%	31	53%	99	81%	510	67%	0.082
	Metachronous	205	35%	27	47%	23	19%	255	33%	
Number of metastatic sites	1	281	48%	31	53%	55	45%	367	48%	0.712
	2	210	36%	18	31%	45	37%	273	36%	
	3–5	94	16%	9	16%	22	18%	125	16%	
Metastatic sites	Liver	378	65%	30	52%	90	74%	498	65%	0.052
	Lung	241	41%	16	28%	48	39%	305	40%	0.044
	Peritoneum	152	26%	24	41%	30	25%	206	27%	0.012
	Lymph nodes	143	24%	17	29%	36	30%	196	26%	0.414
	Bone	22	4%	2	3%	6	5%	30	4%	1.000
	Brain	12	2%	1	2%	3	2%	16	2%	1.000
	Other	64	11%	6	10%	10	8%	80	10%	0.890
ECOG PS	0	220	38%	15	26%	17	14%	252	33%	0.017
	1	189	32%	15	26%	39	32%	243	32%	
	2–4	176	30%	28	48%	65	54%	269	35%	
	Not available	–	–	–	–	1	–	1	–	–
Mutation status	*BRAF*-V600Emt	78	14%	34	59%	11	15%	123	17%	<0.001
	RASmt	320	55%	9	16%	36	49%	365	52%	
	RAS&*BRAF*wt	179	31%	15	26%	26	36%	220	31%	
	Not tested	8	–	–	–	49	–	57	–	–
Type of treatment	Metastasectomy	193	33%	10	17%	10	8%	213	28%	<0.001
	Systemic therapy only	261	45%	21	36%	52	43%	334	44%	
	Best supportive care	131	22%	27	47%	59	49%	217	28%	
	Not known	–	–	–	–	1	–	1	–	–

*dMMR* deficient mismatch repair, *ECOG PS* Eastern Cooperative Oncology Group performance status, *pMMR* proficient mismatch repair

*p-value between pMMR and dMMR.

### Molecular characteristics of the tumours according to a continuum in colon and rectum

When mutation- and MMR-status for primary tumour location were analysed beyond the trichotomous division, *BRAF*-V600Emt were more common from caecum to descending colon compared with sigmoid colon to rectum (27–48% vs 5–6%, *p* < 0.001). dMMR was more prevalent in caecum to the splenic flexure than in descending colon to rectum (15–33% vs 0–2%, *p* < 0.001, Supplementary Table S2).

### Treatments according to clinical and molecular features

In the 510 patients with synchronous disease, the primary tumour was removed in 179 (35%) patients, more often in right and left colon than in rectum (89/187 [48%] vs 60/153 [39%] vs 30/166 [18%], *p* < 0.001). Age did not influence these proportions (data not shown).

Metastasectomy and/or local ablative therapy (LAT) with or without systemic therapy was the treatment for 213 (28%) patients (denoted “metastasectomy”), systemic therapy without metastasectomy for 334 (44%) patients (denoted systemic therapy), and no active tumour controlling therapy besides palliative radiotherapy for the remaining 217 (28%) patients (denoted best supportive care, BSC, Table 1). Metastasectomy rates among actively treated patients was 39% (32% right colon, 41% left colon, 43% rectal cancers (*p* = 0.062).

Patient characteristics according treatment groups are presented in Supplementary Table S3. Actively treated patients were younger than those cared for with BSC and had less right colon and high-grade tumours. The metastasectomy group had right colon primary tumours less often, less synchronous presentation, fewer metastatic sites, and better ECOG-PS compared with the other groups. Patients with *BRAF*-V600Emt tumours had metastasectomies less often than RASmt and RAS&*BRAF*wt (15% vs 31% vs 36%, *p* < 0.001, Table 2). Similarly, patients with dMMR tumours had metastasectomies less often than patients with pMMR tumours (17% vs 33%, *p* < 0.001, Table 3, Fig. 1B).

Chemotherapies according to primary tumour location are presented in Supplementary Table S4. Patients with right colon tumours received fewer treatment lines and more often had palliative intent of first-line chemotherapy compared with left colon and rectal tumours. First-line treatment was, thus, less intensive in right colon primaries, but more similar in further lines.

Chemotherapies according to mutation status are presented in Supplementary Table S5. Patients with *BRAF*-V600Emt tumours received fewer treatment lines, less intensive treatments, and the intent in first line was palliative more often compared with RASmt and RAS&*BRAF*wt. Differences in second-line treatment could also be seen between mutation groups.

Response to first-line chemotherapy was seen less often in *BRAF*-V600Emt compared with RASmt and RAS&*BRAF*wt (partial/complete response in 25% vs 49% vs 56%, *p* < 0.001, Supplementary Table S5). Thus, less responses were seen also in right colon cancers than in left colon and rectal cancers (Supplementary Table S4). No differences were seen in later lines.

In the RAS&*BRAF*wt subgroup a trend was seen for metastasectomies being performed more frequently in patients with rectal primaries compared with right and left colon primaries (Fig. 1, Supplementary Table S6). In this subgroup, number of treatment lines, intent of first-line treatment, or drugs used in different treatment lines did not differ according to primary tumour location. Of 156 patients with RAS&*BRAF*wt tumours treated with chemotherapy, 44 (28%) received an EGFR-inhibitor in first line, and 68 (44%) in later lines.

Among patients receiving chemotherapy, no differences according to MMR-status were seen in number of therapy lines, drugs used in different lines, or response to treatment (Supplementary Table S7). Three of 9 dMMR patients, alive and eligible for immune checkpoint inhibitors after approval, received this treatment and 2 patients received it earlier within a trial [35].

Thirty-seven patients underwent metastasectomies and two had curatively intended LAT without any systemic therapy. Among the patients only receiving BSC, the multifactorial reasons were recorded prospectively; poor ECOG-PS (49%), comorbidity (49%), and high age (40%) were the main reasons.

### Treatment in subgroups according to primary tumour site and mutation and MMR status

Among nine subgroups defined according to primary tumour location and RAS*-*, and *BRAF*-V600E status, metastasectomy rates were the lowest among patients with *BRAF*-V600Emt right colon tumours (12%) and the highest among patients with RAS&*BRAF*wt rectal tumours (42%; Fig. 1A). Systemic therapy only rates varied between the subgroups (range 40–58%). BSC only rates were the lowest among RAS&*BRAF*wt rectal cancers (18%) and the highest among *BRAF*-V600Emt right colon cancers (40%; Fig. 1A).

Among ten subgroups (two <10 patients subgroups excluded) assembled according to primary tumour location and MMR-status, with the pMMR tumours further subdivided by RAS and *BRAF* status, the metastasectomy rates varied from 9% in *BRAF*-V600Emt/pMMR rectal cancers to 43% in RAS&*BRAF*wt/pMMR rectal cancers (Fig. 1B). Systemic therapy only rates varied less between the subgroups (range 41–64%). BSC rates were the lowest among RAS&*BRAF*wt/pMMR rectal cancers (15%) and the highest among dMMR right colon cancers (46%).

### Fitness and eligibility for intensive therapy according to primary tumour location, mutation status and MMR-status

When retrospectively studying fitness for intensive therapy according to patient characteristics, based on ESMO-guidelines (exclusion criteria age >75 years and ECOG-PS 2–4, [2]), 44% were considered “fit for intensive therapy” (Supplementary Table S8A). This proportion varied widely according to primary tumour location, mutation status, and MMR-status, being lowest among *BRAF*-V600Emt and dMMR.

Of all, 36% were considered “eligible for intensive systemic therapy” (exclusion criteria unfit as above, receiving only one drug in one treatment line, or curative metastasectomy without perioperative therapy, and BSC only, Supplementary Table S8B). The highest eligibility of almost 50% was seen in rectal cancers, and the lowest 21% for dMMR patients.

### Overall survival in all patients and according to presentation and treatment type

Among all patients mOS from diagnosis of metastatic disease was 15.1 months (95% CI 13.1–17.1). Patients with metachronous metastases did better than patients with synchronous metastases (mOS 20.4 vs 13.2 months, HR: 0.81 [95% CI 0.68–0.95], Supplementary Fig. S1A), being most evident in patients with rectal tumours (Supplementary Fig. S1B–D).

In actively treated patients, mOS was 23.0 months (95% CI 20.6–25.4). The best mOS was seen in those treated with metastasectomy, followed by systemic therapy only, and the shortest in the BSC group (mOS 63.2 vs 13.8 vs 4.3 months; HR: 0.18 [95% CI 0.14–0.23], reference, 2.60 [95% CI 2.18–3.11]; Supplementary Fig. S2A). These results withstood in conditional landmark analyses at 4 months and 3 weeks (Supplementary Fig. S2B, C).

### Overall survival according to primary tumour location, mutation status, and mismatch repair status

**Fig. 2:**
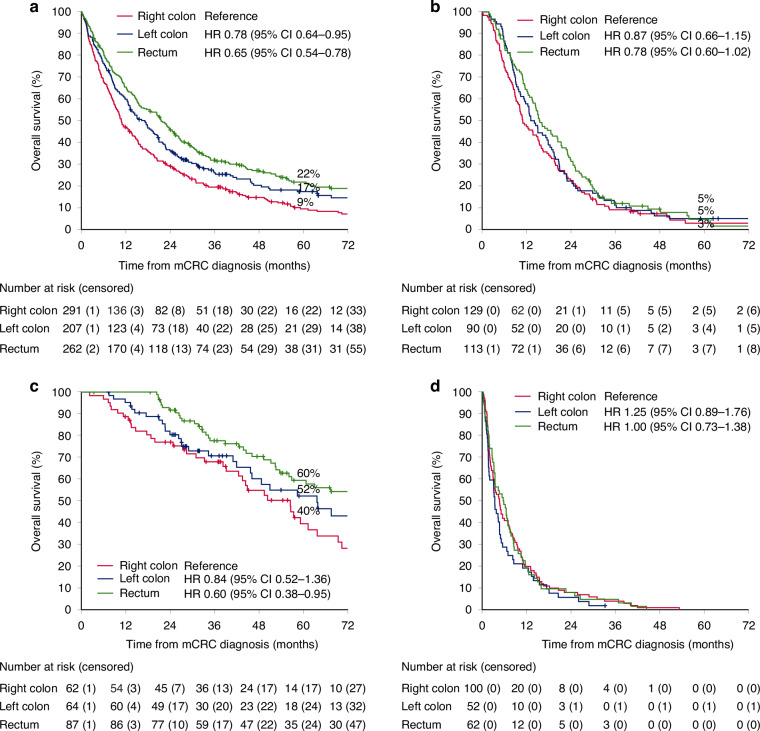
Overall survival according to primary tumour location. For all patients (**a**), and separately for patients treated with systemic therapy only (**b**), metastasectomy (**c**), and best supportive care only (**d**).

Patients with right colon tumours had worse OS than patients with left colon and rectal tumours (mOS 10.8 vs 17.2 vs 21.6 months; Fig. 2A). When stratified by treatment, no statistically significant differences were seen between right and left colon primaries in any treatment group (Fig. 2B–D). In the metastasectomy group, rectal cancers did better than the other two locations (Fig. 2C). These results withstood in a conditional landmark analysis at 4 months (Supplementary Fig. S3).

**Fig. 3:**
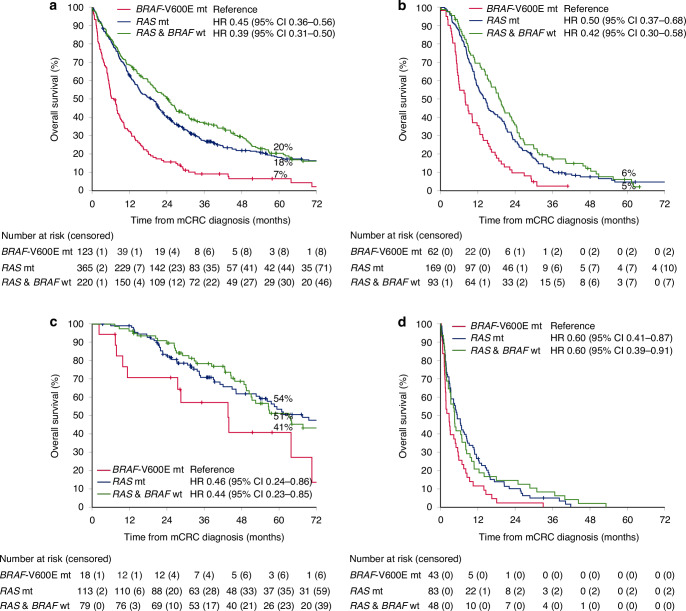
Overall survival according to mutation status. For all patients (**a**), and separately for patients treated with systemic therapy only (**b**), metastasectomy (**c**), and best supportive care only (**d**).

Patients with *BRAF*-V600Emt tumours had an inferior OS compared with those that had RASmt or RAS&*BRAF*wt tumours (mOS 6.9 vs 19.0 vs 24.4 months; Fig. 3A). These differences remained statistically significant in analyses stratified by treatment (Fig. 3B–D).

*PIK3CA*mt status did not influence OS, neither in all patients (Supplementary Fig. S4), nor in analyses stratified by primary tumour location, RAS/*BRAF*-V600E mutation status, or treatment groups (data not shown).

**Fig. 4:**
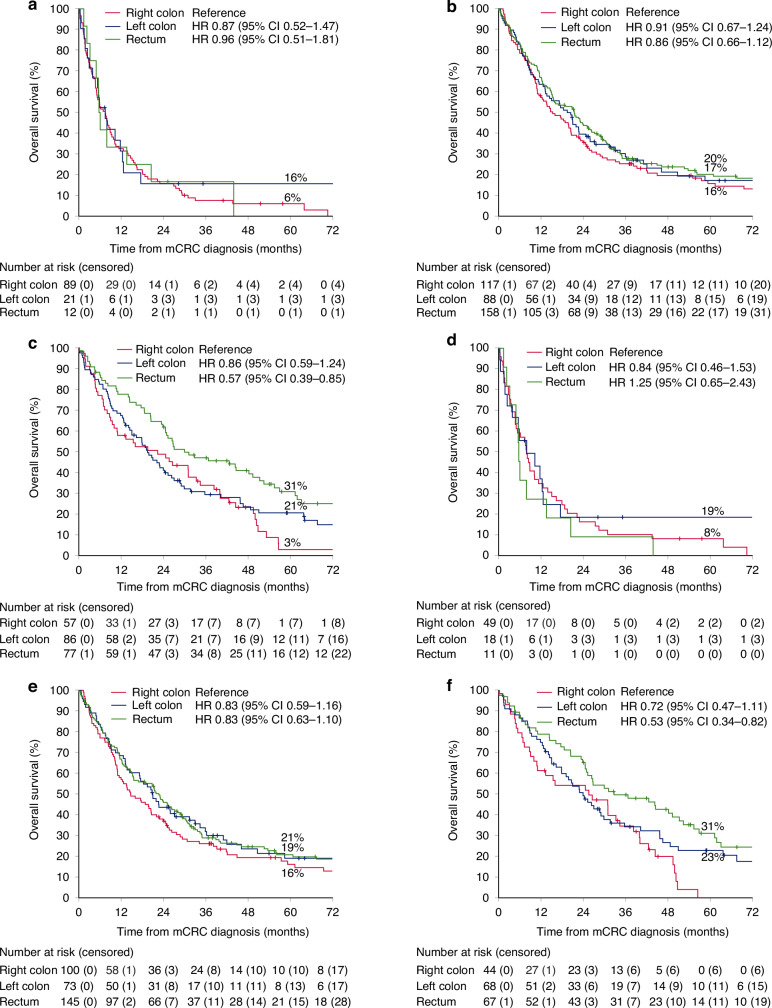
Overall survival according to primary tumour location in molecular subgroups. According to mutation status; *BRAF*-V600Emt (**a**), RASmt (**b**), and RAS&*BRAF*wt (**c**), and according to mutation status in patients with proficient mismatch repair; *BRAF*-V600Emt (**d**), RASmt (**e**), and RAS&*BRAF*wt (**f**).

OS for all tested patients according to primary tumour location stratified by mutation status is presented in Fig. 4. No OS differences were seen between primary tumour locations in the *BRAF*-V600Emt and RASmt subgroups (Fig. 4A, B). In the RAS&*BRAF*wt subgroup, patients with rectal primary tumours had the best OS, whereas no survival difference was seen between patients with right and left colon tumours (Fig. 4C). This was also seen when analysed separately in pMMR patients (Fig. 4D, F). Among RAS&*BRAF*wt and pMMR patients, no differences in OS were seen for systemic therapy or BSC only patients, whereas rectal cancers did the best in the metastasectomy group, with caveat of small subgroups (Supplementary Fig. S5A–C).

When studying OS for primary tumour location divided beyond the trichotomous division, some heterogeneity was observed between right colon locations (mOS 8.2–12.0 months), whereas greater variability was observed in subgroups of left colon with splenic flexure having shorter OS than descending colon and sigmoid colon (mOS 10.8 vs 17.2 vs 17.9 months), while rectum still had the best OS (mOS 21.6 months, Supplementary Fig. S6A). No OS differences were seen in the *BRAF*-V600Emt and RASmt subgroups (Supplementary Fig. S6B, C), whereas small differences were seen in the RAS&*BRAF*wt subgroup (Supplementary Fig. S6D), again with caveat of small subgroups.

Among nine subgroups defined according to primary tumour location, and RAS*-* and *BRAF-*status, mOS after metastasectomy was the shortest among patients with *BRAF*-V600Emt right colon cancers (29 months) and the longest among patients with RAS&*BRAF*wt rectal cancers (95 months; Fig. 1, Supplementary Fig. S7A–I). Among patients receiving systemic therapy only, mOS varied from 6 months in patients with *BRAF*-V600Emt left colon and rectal cancers to 23 months in patients with RAS&*BRAF*wt rectal cancers. In the BSC only group, mOS varied from 3 to 7 months, without significant differences between subgroups.

Patients with dMMR tumours had significantly worse OS than those with pMMR tumours (mOS 13.8 vs 22.9 months; Supplementary Fig. S8A). However, there were no OS differences between dMMR and pMMR patients in analyses stratified by treatment or mutational status (Supplementary Figs. S8B–D,  S9A–C). Among five patients treated with checkpoint inhibitors, mOS was 64.0 months (OS 25+, 29, 30+, 47+, and 64 months).

### Overall survival according to primary tumour location, mismatch repair status, mutation status, and treatment type

In ten subgroups assembled according to primary tumour location and MMR-status, with pMMR tumours further subdivided by RAS/*BRAF* mutation status, the shortest OS after metastasectomies was seen in patients in the RAS&*BRAF*wt/pMMR right colon subgroup (44 months) and the longest in patients with RAS&*BRAF*wt/pMMR rectal cancers (93 months; Fig. 1B). In the subgroups receiving systemic therapy only, the shortest mOS was seen among *BRAF*-V600Emt/pMMR rectal cancers (6 months) and the longest among RAS&*BRAF*wt/pMMR rectal cancers (23 months). BSC subgroups did poorly with mOS varying between 1 and 9 months.

### Univariable and multivariable overall survival analyses

In the multivariable model, patients with *BRAF*-V600Emt tumours demonstrated a worse OS than patients with RASmt and RAS&*BRAF*wt tumours (Table 4). Number of metastatic sites, ECOG-PS, tumour grade, and type of treatment also remained statistically significant. Age, presentation of metastases, and MMR-status remained statistically significant, however, in an opposite direction, whereas interlinked primary tumour location turned out non-significant.

**Table 4:** Univariable and multivariable Cox regression models of factors affecting overall survival.

			Univariable			Multivariable		
		*N*	HR	95% CI	*p*-value	HR	95% CI	*p*-value
Age		764	1.03	1.02–1.04	<0.001	0.99	0.98–1.00	0.009
Primary tumour location	Right colon	291	1			1		
	Left colon	206	0.78	0.64–0.95	0.012	1.08	0.87–1.34	0.475
	Rectum	262	0.65	0.54–0.78	<0.001	1.00	0.81–1.22	0.972
	Unknown	5	2.18	0.81–5.87	0.123	1.87	0.69–5.09	0.222
Tumour grade	Low	466	1			1		
	High	216	1.87	1.57–2.22	<0.001	1.57	1.30–1.90	<0.001
	Not available	82	2.52	1.97–3.22	<0.001	1.56	1.19–2.04	0.001
Presentation of metastases	Synchronous	509	1			1		
	Metachronous	255	0.81	0.68–0.95	0.012	1.20	1.00–1.44	0.050
Number of metastatic sites	1	366	1			1		
	2	273	1.65	1.36–1.93	<0.001	1.28	1.07–1.54	0.008
	3–5	125	2.09	1.68–2.60	<0.001	1.51	1.20–1.90	<0.001
ECOG PS^a^	0	252	1			1		
	1	243	2.57	2.09–3.17	<0.001	1.95	1.56–2.43	<0.001
	2–4	269	6.65	5.38–8.21	<0.001	2.85	2.23–3.65	<0.001
Type of treatment^a^	Systemic therapy only	334	1			1		
	Metastasectomy	213	0.18	0.14–0.23	<0.001	0.23	0.18–0.30	<0.001
	Best supportive care	217	2.60	2.18–3.11	<0.001	2.56	2.01–3.27	<0.001
Mutation groups	*BRAF*-V600Emt	123	1			1		
	RASmt	365	0.46	0.37–0.57	<0.001	0.48	0.38–0.62	<0.001
	RAS&*BRAF*wt	220	0.40	0.31–0.52	<0.001	0.43	0.33–0.56	<0.001
	Not tested	56	1.48	1.07–2.05	0.018	0.67	0.44–1.00	0.051
MMR status	pMMR	585	1			1		
	dMMR	58	1.43	1.07–1.92	0.016	0.51	0.36–0.70	<0.001
	Not tested	121	2.02	1.64–2.50	<0.001	1.14	0.88–1.48	0.307

*dMMR* deficient mismatch repair, *ECOG PS* European Cooperative Oncology Group performance status, *MMR* mismatch repair, *pMMR* proficient mismatch repair.

^a^1 with missing ECOG PS and treatment information excluded.

## Discussion

This recently collected cohort of mCRC patients from a defined population, where in practice 100% of all cases diagnosed were identified, shows that certain molecular subgroups differ markedly in frequency from clinical trials/hospital-based series when all patients, i.e., also non-actively treated, BSC only, patients are included. Thus, higher proportions had *BRAF*-V600Emt (17%) and dMMR (9%) in this cohort compared with 4–12% and 3–5%, respectively, in clinical trials [36–40]. Patients with these characteristics are generally less fit and, thus, less often make it to a trial and are less often treated at hospitals reporting data in research publications. We could also show that more tumours were right-sided (38%) than reported in recent clinical trials (25–30%) [41–43] and confirm that right colon tumours have worse survival than left colon and rectal tumours [42, 44].

In line with previous studies [2, 11–13], both *BRAF*-V600Emt and dMMR were more common in right colon tumours than in left colon or rectal tumours. RASmt are more frequent in rectal cancers but seen throughout colon and rectum, and thus, much fewer patients with right colon tumours have RAS&*BRAF*wt tumours, in this cohort, only 22% compared with 44% for left colon tumours and 31% for rectal tumours (*p* < 0.001). When also MMR-status was considered (56% overlap between dMMR and *BRAF*-V600Emt), the differences became even more marked (RAS&*BRAF*wt/pMMR in 18% vs 41% vs 30%, *p* < 0.001), i.e., only a fraction of right colon tumours belong to a “treatment-sensitive group”. If *PIK3CA*mt also is included in the EGFR-inhibitor non-sensitive group [27, 45], the gap becomes even larger (RAS&*BRAF*wt/ pMMR/*PIK3CA*wt in 15% vs 41% vs 27%, *p* < 0.001).

When mutation status and MMR-status were studied in the CRC continuum, the higher prevalence of *BRAF*-V600Emt and dMMR, usually seen in right-sided tumours, continued beyond the transverse colon, an aspect also reported before [21, 23]. These findings indicate that dividing the colorectum into two or three parts might be too coarse for some applications [20], for example when predicting response to EGFR-inhibitors [2, 27]. Although numbers were limited in some subgroups, *BRAF*-V600Emt and dMMR were more common in ascending colon than in caecum, as also reported [23].

The OS differences according to primary tumour location and molecular subgroups were seen in the actively treated groups, but interestingly also in the BSC group, for *BRAF*-V600Emt. In virtually all reports about outcomes in mCRC patients in literature, patients have been actively treated [15, 17–19, 38, 43, 46]. Metastasectomy rates were lower with 21% in right colon primary tumours compared with 31% for left colon and 33% for rectum and their survival worse, in line with previous findings [16, 46, 47], whereas in actively treated patients, the proportions were more similar, or 32% vs 42% vs 44%, respectively. We show differences in metastatic sites for right colon primary tumour location with less liver and lung metastases and more peritoneal metastases, poorer ECOG-PS, older age, and more dMMR and *BRAF*mt tumours with a more aggressive clinical course. The wide variability in metastasectomy rates (13–43%) and survival (mOS 44–93 months) according to primary tumour location, MMR-status, and mutation status are most probably explained by the differences in RAS*-*, *BRAF-*, and MMR-status which, thus, seem most important. This aspect of combining primary tumour location, mutation status, and MMR-status has not previously been analysed in detail.

The systemic therapy only frequency was 41–64% according to primary tumour location, RAS-, *BRAF-*, and MMR-status, with a markedly variable mOS of 6–23 months, which cannot be explained by the minor differences in systemic therapy intensity in the subgroups. These outcomes contrast highly to the best mOS of 30–40 months in selected, RAS&*BRAF*wt and pMMR patients in recent clinical trials [2, 3, 43]. The BSC rate also varied highly 15–46%, and mOS was only a few months, in line with previous studies [5, 11].

Of all patients, 44% were fit for the most intensive therapy based on age ≤75 years and ECOG-PS 0-1 per ESMO-guidelines [2]. We are aware that this is a too crude way of defining fitness, but it has been used for example in triplet chemotherapy studies [2], and is a surrogate for significant co-morbidity, not recorded well enough to be used here. The proportion of ≤75-year-old not fit for intensive therapy is probably reasonably comparable to the proportion of >75-year-old fit for intensive therapy.

If the actual treatments given were considered, only about one-third or 36% were eligible for intensive therapy. Eleven percent were treated and potentially “cured” with metastasectomy only without receiving perioperative systemic therapy, 7% received only a single drug in the first-line situation, and 28% received BSC. A large proportion (94%) of those eligible for intensive therapy received combination chemotherapy with or without biologics (data not shown).

The comparisons of OS in homogenous mutation groups, even if hampered by few individuals in some subgroups, indicate that the relevance of primary tumour location is in large due to differences in clinical characteristics, metastatic sites, mutational- and MMR-status, and consequently, treatment allocation. When comparing primary tumour location, no significant differences in OS for patients with RASmt or *BRAF*-V600Emt tumours were noted, as also reported by others [48, 49]. When comparing patients with primary tumours in right and left colon, we saw no difference in OS, contrary to another study showing a worse prognosis for right colon tumours in the *BRAF*wt group [48]. Rectal RAS&*BRAF*wt tumours, however, had a superior OS compared with right colon RAS&*BRAF*wt tumours, which was also observed in the pMMR subset, this highlights the importance of analysing rectal cancers separately and not lumping them together with the other left-sided tumours.

In a multivariable analysis, primary tumour location was nonsignificant when adjusting for other factors including treatment. Contrary to our results, there was a difference in OS between right- and left-sided primaries in multivariable analyses of an Australian register-based study and between right and left colon in the PETACC3 mCRC subgroup [48, 50]. These two studies did, however, not separate right colon, left colon, and rectum and the study populations were selected.

The highest BSC only rate (46%) and ineligibility for intensive therapy (79%, with caveat that some >75-year-old may tolerate immune checkpoint inhibitors) was seen among dMMR tumours, a trend also seen in [13]. The prognosis of treatable dMMR tumours will markedly change in the future due to immune checkpoint inhibitors [35]. This could be seen already in this study (Supplementary Fig. S7B) even if these drugs were primarily used after approval in 2021 and only 5 dMMR patients received this treatment with impressive OS. A similar improvement will probably be seen also in other subgroups in the future after the introduction of other targeted drugs, for example the combination of encorafenib-cetuximab and chemotherapy [51].

The relevance of a particular treatment, for example EGFR-inhibition is best explored in randomised clinical trials [52]. In population-based cohorts, such as this study, heterogeneity is always present. Too few individuals were treated with an EGFR-inhibitor in first line to allow for comparisons according to tumour location. Interestingly, only 16% of population-based left-sided cancers were RAS&*BRAF*wt, pMMR, and eligible for intensive therapy. EGFR-inhibitors in any line were used in approximately 80% of patients eligible to intensive therapy, irrespective of sidedness in RAS&*BRAF* wild-type tumours in line with the Swedish guidelines [2, 3]. Recent trials comparing EGFR-inhibitors versus vascular endothelial growth factor inhibitors in combination with chemotherapy in RASwt mCRC have indicated benefit from EGFR-inhibitors in left-sided cancers but not in right-sided cancers, albeit not universally [17, 43, 46, 52].

*PIK3CA*mt was more common in right colon than in left colon and rectum, also reported by others [12, 53, 54]. In our cohort, *PIK3CA*mt did not affect OS, contrary to a meta-analysis indicating a worse OS after EGFR-inhibitor therapy [55]. The different distribution of *PIK3CA*mt according to primary tumour location and their relative resistance to EGFR-inhibitors [27], may partially explain survival differences according to primary tumour location.

### Strengths and limitations

The strength is that the patient material is truly population-based without any selection meaning that the clinical presentation, treatments provided, and OS reflect a real-world situation where metastasectomies have been frequently used. Allocation to treatment groups and chemotherapies used were very reliably recorded making it possible to study these factors in detail. A very high 93% rate of molecularly analysed samples was achieved as all patients with sufficient material were analysed. Thus, the molecular analyses are representative also for the group of patients cared for with BSC, usually not tested or included in other studies.

However, sufficient tumour material for analyses is not always present (7% in this cohort) meaning that these results do not fully reflect the true incidence. Biopsies were sometimes too small or used up in clinical routine. We have previously noted that this drop-out most probably means that the groups with the poorest prognosis, *BRAF*-V600Emt and dMMR in reality are slightly higher than noted here, further increasing the gap to materials from clinical trials/hospital-based series [11, 12].

A further weakness is that different methods were used for the molecular analyses. Some analyses were done in clinical routine, whereas others were done for the purpose of this study, though with established methods. Preferably, MMR should have been analysed either using PCR or IHC but using TMB as a proxy to define MMR-status has been deemed sufficiently reliable [33], allowing for another 20% to be characterised for MMR. Congruence for RAS/*BRAF*/*PIK3CA* was usually seen between cases analysed by clinical routine and with the Oncomine/WGS platforms but differed for 31 cases (5%), the choice of another sample with lower tumour cell count, or methodological issues, are possible reasons.

## Conclusions

This population-based cohort shows that primary tumour location, RAS/*BRAF*-status, and MMR-status affect clinical characteristics, treatability, and outcome of mCRC. High metastasectomy rates of 39% were achieved population-based among actively treated, but not all actively treated were fit for intensive systemic treatment and 28% received BSC only. Our data show that *BRAF*-V600Emt and dMMR are more common population-based and in right colon tumours, have fewer metastasectomies and more BSC only, and worse OS. This could explain the worse OS seen in right colon tumours, which is supported by the minor OS differences between right colon and left colon primary tumours in analyses stratified by treatment and mutation status, and that primary tumour location was non-significant in multivariable analyses. Therefore, we propose that molecular alterations are more important to consider than primary tumour location when it comes to evaluation of prognosis both overall and in treatment groups.

## Supplementary information

Supplementary material

## Data Availability

The data that support the findings of this study are available from the corresponding author upon reasonable request, with approval by the steering committee.
