# Supplementary material for: Primary tumour location, molecular alterations, treatments, and outcome in a population-based metastatic colorectal cancer cohort
Source: BJC Rep. 2025 May 28;3:38. doi: 10.1038/s44276-025-00156-z (PMC12120107; doi:10.1038/s44276-025-00156-z)
Supplement: Supplementary file 1 — Supplementary material [file 44276_2025_156_MOESM1_ESM.docx]

Emerik Osterlund, Klara Hammarström, Luís Nunes, Lucy Mathot, Artur Mezheyeuski, Tobias Sjöblom, and Bengt Glimelius.
**Supplementary material for: Primary tumour location, molecular alterations, treatment, and outcome in an unselected population of metastatic colorectal cancer patients**

**Table S1. Patient characteristics according to if molecular testing was done or not**

|  |  | Molecularly tested | | Not tested | | Total* | | p-value |
| --- | --- | --- | --- | --- | --- | --- | --- | --- |
|  |  | 708 | 93 % | 57 | 7 % | 765 | 100 % |  |
| Median age (range) |  | 72 (28-99) | | 78 (56-96) | | 72 (28-99) | | <0.001 |
| Total |  | 708 | 100 % | 57 | 100 % | 765 | 100 % | <0.001 |
| Age groups | ≤70 years | 302 | 43 % | 11 | 19 % | 313 | 41 % |  |
|  | >70 years | 406 | 57 % | 46 | 81 % | 452 | 59 % |  |
| Sex | Male | 375 | 53 % | 30 | 53 % | 405 | 53 % | 0.961 |
|  | Female | 333 | 47 % | 27 | 47 % | 360 | 47 % |  |
| Primary tumour | Right colon | 263 | 37 % | 28 | 51 % | 291 | 38 % | 0.136 |
| location | Left colon | 195 | 28 % | 12 | 22 % | 207 | 27 % |  |
|  | Rectum | 247 | 35 % | 15 | 27 % | 262 | 34 % |  |
|  | Unknown/multiple | 3 | - | 2 | - | 5 | - | - |
| Primary resection | No | 292 | 41 % | 47 | 82 % | 339 | 44 % | <0.001 |
|  | Yes | 416 | 59 % | 10 | 18 % | 426 | 56 % |  |
| Tumour grade | Low | 445 | 69 % | 22 | 55 % | 467 | 68 % | 0.061 |
|  | High | 198 | 31 % | 18 | 45 % | 216 | 32 % |  |
|  | Missing | 65 | - | 17 | - | 82 | - | - |
| Presentation of | Synchronous | 460 | 65 % | 50 | 88 % | 510 | 67 % | <0.001 |
| metastases | Metachronous | 248 | 35 % | 7 | 12 % | 255 | 33 % |  |
| Number of | 1 | 338 | 48 % | 29 | 51 % | 367 | 48 % | 0.686 |
| metastatic sites | 2 | 252 | 36 % | 21 | 37 % | 273 | 36 % |  |
|  | 3-5 | 118 | 17 % | 7 | 12 % | 125 | 16 % |  |
| Metastatic sites | Liver | 456 | 64 % | 42 | 74 % | 498 | 65 % | 0.157 |
|  | Lung | 287 | 41 % | 18 | 32 % | 305 | 40 % | 0.184 |
|  | Peritoneum | 196 | 28 % | 10 | 18 % | 206 | 27 % | 0.097 |
|  | Lymph nodes | 177 | 25 % | 19 | 33 % | 196 | 26 % | 0.166 |
|  | Bone | 29 | 4 % | 1 | 2 % | 30 | 4 % | 0.719 |
|  | Brain | 14 | 2 % | 2 | 4 % | 16 | 2 % | 0.338 |
|  | Other | 78 | 11 % | 2 | 4 % | 80 | 10 % | 0.075 |
| ECOG PS | 0 | 250 | 35 % | 2 | 4 % | 252 | 33 % | <0.001 |
|  | 1 | 222 | 31 % | 21 | 38 % | 243 | 32 % |  |
|  | 2-4 | 236 | 33 % | 33 | 59 % | 269 | 35 % |  |
|  | Missing | - | - | 1 | - | 1 | - | - |
| Type of treatment | Metastasectomy | 210 | 30 % | 3 | 5 % | 213 | 28 % | <0.001 |
|  | Systemic therapy only | 324 | 46 % | 10 | 18 % | 334 | 44 % |  |
|  | Best supportive care | 174 | 25 % | 43 | 77 % | 217 | 28 % |  |
|  | Not known | - | - | 1 | - | 1 | - | - |
| ECOG PS=Eastern Cooperative Oncology Group performance status | | | | | | | | |

**Table S2. Mutation status and mismatch repair status in the colorectal cancer continuum**

|  |  | Caecum | | Ascending colon | | Hepatic flexure | | Transverse colon | | Splenic flexure | | Descending colon | | Sigmoid colon | | Rectum | | Total* | | p-value |
| --- | --- | --- | --- | --- | --- | --- | --- | --- | --- | --- | --- | --- | --- | --- | --- | --- | --- | --- | --- | --- |
|  |  | 149 | 19 % | 68 | 9 % | 34 | 4 % | 40 | 5 % | 27 | 4 % | 20 | 3 % | 160 | 21 % | 262 | 34 % | 765 | 100 % |  |
| Mutation status | *BRAF*-V600Emt | 36 | 27 % | 29 | 48 % | 9 | 28 % | 15 | 42 % | 6 | 24 % | 6 | 32 % | 9 | 6 % | 12 | 5 % | 123 | 17 % | <0.001 |
|  | *RAS*mt | 75 | 56 % | 22 | 36 % | 11 | 34 % | 9 | 25 % | 9 | 36 % | 6 | 32 % | 73 | 48 % | 158 | 64 % | 365 | 52 % |  |
|  | *RAS*&*BRAF*wt | 23 | 17 % | 10 | 16 % | 12 | 38 % | 12 | 33 % | 10 | 40 % | 7 | 37 % | 69 | 46 % | 77 | 31 % | 220 | 31 % |  |
|  | Not tested | 15 | - | 7 | - | 2 | - | 4 | - | 2 | - | 1 | - | 9 | - | 15 | - | 57 | - | - |
| MMR-status | pMMR | 106 | 85 % | 38 | 67 % | 26 | 84 % | 29 | 83 % | 15 | 75 % | 16 | 100 % | 128 | 98 % | 225 | 99 % | 438 | 90 % | <0.001 |
|  | dMMR | 18 | 15 % | 19 | 33 % | 5 | 16 % | 6 | 17 % | 5 | 25 % | 0 | 0 % | 2 | 2 % | 3 | 1 % | 50 | 10 % |  |
|  | Not tested | 25 | - | 11 | - | 3 | - | 5 | - | 7 | - | 4 | - | 30 | - | 34 | - | 277 | - | - |
| *5 with unknown/multiple primary tumours not presented separately, dMMR=deficient mismatch repair, MMR=mismatch repair, pMMR=proficient mismatch repair | | | | | | | | | | | | | | | | | | | | |

**Table S3. Patient characteristics according to treatment groups**

|  |  | Metastasectomy  +/- systemic therapy | | Systemic therapy only | | Best supportive care | | Total* | | p-value |
| --- | --- | --- | --- | --- | --- | --- | --- | --- | --- | --- |
|  |  | 213 | 28 % | 334 | 44 % | 217 | 28 % | 765 | 100 % |  |
| Median age (range) | | 68 (35-89) | | 70 (28-89) | | 82 (48-99) | | 72 (28-99) | | <0.001 |
| Total |  | 213 | 100 % | 334 | 100 % | 217 | 100 % | 765 | 100 % |  |
| Age groups | ≤70 years | 121 | 57 % | 163 | 49 % | 28 | 13 % | 313 | 41 % | <0.001 |
|  | >70 years | 92 | 43 % | 171 | 51 % | 189 | 87 % | 452 | 59 % |  |
| Sex | Male | 111 | 52 % | 182 | 54 % | 111 | 51 % | 405 | 53 % | 0.720 |
|  | Female | 102 | 48 % | 152 | 46 % | 106 | 49 % | 360 | 47 % |  |
| Primary tumour | Right colon | 62 | 29 % | 129 | 39 % | 100 | 47 % | 291 | 38 % | 0.006 |
| location | Left colon | 64 | 30 % | 90 | 27 % | 52 | 24 % | 207 | 27 % |  |
|  | Rectum | 87 | 41 % | 113 | 34 % | 62 | 29 % | 262 | 34 % |  |
|  | Unknown | - | - | 2 | - | 3 | - | 5 | - | - |
| Primary resection | No | 18 | 8 % | 208 | 62 % | 113 | 52 % | 339 | 44 % | <0.001 |
|  | Yes | 195 | 92 % | 126 | 38 % | 104 | 48 % | 426 | 56 % |  |
| Tumour grade | Low | 174 | 83 % | 178 | 62 % | 114 | 62 % | 467 | 68 % | <0.001 |
|  | High | 36 | 17 % | 110 | 38 % | 70 | 38 % | 216 | 32 % |  |
|  | Missing | 3 | - | 46 | - | 33 | - | 82 | - | - |
| Presentation of | Synchronous | 113 | 53 % | 258 | 77 % | 138 | 64 % | 510 | 67 % | <0.001 |
| metastases | Metachronous | 100 | 47 % | 76 | 23 % | 79 | 36 % | 255 | 33 % |  |
| Number of | 1 | 147 | 69 % | 115 | 34 % | 104 | 48 % | 367 | 48 % | <0.001 |
| metastatic sites | 2 | 52 | 24 % | 134 | 40 % | 87 | 40 % | 273 | 36 % |  |
|  | 3-5 | 14 | 7 % | 85 | 25 % | 26 | 12 % | 125 | 16 % |  |
| Metastatic sites | Liver | 139 | 65 % | 231 | 69 % | 127 | 59 % | 498 | 65 % | 0.038 |
|  | Lung | 63 | 30 % | 162 | 49 % | 80 | 37 % | 305 | 40 % | <0.001 |
|  | Peritoneum | 47 | 22 % | 93 | 28 % | 66 | 30 % | 206 | 27 % | 0.133 |
|  | Lymph nodes | 22 | 10 % | 126 | 38 % | 48 | 22 % | 196 | 26 % | <0.001 |
|  | Bone | 1 | 0 % | 20 | 6 % | 9 | 4 % | 30 | 4 % | 0.002 |
|  | Brain | 3 | 1 % | 7 | 2 % | 6 | 3 % | 16 | 2 % | 0.657 |
|  | Other | 21 | 10 % | 31 | 9 % | 28 | 13 % | 80 | 10 % | 0.376 |
| ECOG PS | 0 | 142 | 67 % | 103 | 31 % | 7 | 3 % | 252 | 33 % | <0.001 |
|  | 1 | 59 | 28 % | 135 | 40 % | 49 | 23 % | 243 | 32 % |  |
|  | 2-4 | 12 | 6 % | 96 | 29 % | 161 | 74 % | 269 | 35 % |  |
|  | Not available | - | - | - | - | - | - | 1 | - | - |
| Mutation status | RAS&*BRAF*wt | 79 | 38 % | 93 | 29 % | 48 | 28 % | 220 | 31 % | <0.001 |
|  | RASmt | 113 | 54 % | 169 | 52 % | 83 | 48 % | 365 | 52 % |  |
|  | *BRAF*-V600Emt | 18 | 9 % | 62 | 19 % | 43 | 25 % | 123 | 17 % |  |
|  | Not tested | 3 | - | 10 | - | 43 | - | 57 | - | - |
| MMR-status | pMMR | 163 | 95 % | 184 | 92 % | 91 | 79 % | 438 | 90 % | <0.001 |
|  | dMMR | 9 | 5 % | 17 | 8 % | 24 | 21 % | 50 | 10 % |  |
|  | Not tested | 41 | - | 133 | - | 102 | - | 277 | - | - |
| *1 with unknown treatment not presented separately, dMMR=deficient mismatch repair, ECOG PS=Eastern Cooperative Oncology Group performance status, MMR=mismatch repair, pMMR=proficient mismatch repair | | | | | | | | | | |

**Table S4. Systemic therapy according to primary tumour location**

|  |  | Right colon | | Left colon | | Rectum | | Total* | | p-value |
| --- | --- | --- | --- | --- | --- | --- | --- | --- | --- | --- |
|  |  | 181 | 36 % | 142 | 28 % | 183 | 36 % | 508 | 100 % |  |
| Number of treatment | 1 | 92 | 51 % | 55 | 39 % | 75 | 41 % | 223 | 44 % | 0.031 |
| lines | 2 | 50 | 28 % | 50 | 35 % | 47 | 26 % | 148 | 29 % |  |
|  | 3+ | 39 | 22 % | 37 | 26 % | 61 | 33 % | 137 | 27 % |  |
| First line intent | Palliative | 136 | 75 % | 91 | 64 % | 120 | 66 % | 349 | 69 % | 0.021 |
|  | Conversion | 12 | 7 % | 26 | 18 % | 29 | 16 % | 67 | 13 % |  |
|  | Neo-adjuvant | 21 | 12 % | 18 | 13 % | 23 | 13 % | 62 | 12 % |  |
|  | Adjuvant | 12 | 7 % | 7 | 5 % | 11 | 6 % | 30 | 6 % |  |
| First line treatment | Single | 41 | 23 % | 26 | 18 % | 32 | 17 % | 100 | 20 % | 0.039 |
|  | Single+biologic | 19 | 10 % | 9 | 6 % | 14 | 8 % | 42 | 8 % |  |
|  | Doublet | 90 | 50 % | 71 | 50 % | 77 | 42 % | 239 | 47 % |  |
|  | Doublet+biologic | 30 | 17 % | 25 | 18 % | 47 | 26 % | 102 | 20 % |  |
|  | Triplet±biologic | 0 | 0 % | 10 | 7 % | 11 | 6 % | 21 | 4 % |  |
|  | Other | 1 | 1 % | 1 | 1 % | 2 | 1 % | 4 | 1 % | - |
| Second line treatment | Single | 8 | 9 % | 10 | 11 % | 5 | 5 % | 23 | 8 % | 0.052 |
|  | Single+biologic | 16 | 18 % | 24 | 28 % | 20 | 19 % | 60 | 21 % |  |
|  | Doublet | 40 | 45 % | 29 | 33 % | 39 | 36 % | 109 | 38 % |  |
|  | Doublet+biologic | 18 | 20 % | 22 | 25 % | 41 | 38 % | 81 | 28 % |  |
|  | Triplet±biologic | 0 | 0 % | 1 | 1 % | 0 | 0 % | 1 | 0 % |  |
|  | Other | 7 | 8 % | 1 | 1 % | 3 | 3 % | 11 | 4 % | - |
| Third line treatment | Single | 2 | 5 % | 4 | 11 % | 7 | 11 % | 13 | 9 % | 0.330 |
|  | Single+biologic | 13 | 33 % | 8 | 22 % | 11 | 18 % | 32 | 23 % |  |
|  | Doublet | 5 | 13 % | 9 | 24 % | 18 | 29 % | 32 | 23 % |  |
|  | Doublet+biologic | 10 | 26 % | 7 | 19 % | 12 | 19 % | 29 | 21 % |  |
|  | Other | 9 | 23 % | 9 | 24 % | 14 | 23 % | 32 | 23 % | - |
| Fourth line treatment | Single | 0 | 0 % | 1 | 8 % | 0 | 0 % | 1 | 2 % | N/A |
|  | Single+biologic | 2 | 15 % | 2 | 15 % | 2 | 10 % | 6 | 13 % |  |
|  | Doublet | 2 | 15 % | 1 | 8 % | 3 | 14 % | 6 | 13 % |  |
|  | Doublet+biologic | 4 | 31 % | 1 | 8 % | 6 | 29 % | 11 | 23 % |  |
|  | Other | 5 | 38 % | 8 | 62 % | 10 | 48 % | 23 | 49 % | - |
| Response first line | Partial/complete response | 45 | 35 % | 50 | 46 % | 82 | 55 % | 177 | 46 % | 0.013 |
|  | Stable disease | 40 | 31 % | 27 | 25 % | 36 | 24 % | 103 | 27 % |  |
|  | Progressive disease | 44 | 34 % | 31 | 29 % | 30 | 20 % | 107 | 28 % |  |
|  | Not available | 52 | - | 34 | - | 35 | - | 121 | - | - |
| Response second line | Partial/complete response | 14 | 21 % | 26 | 35 % | 28 | 30 % | 68 | 29 % | 0.118 |
|  | Stable disease | 27 | 40 % | 18 | 24 % | 37 | 39 % | 82 | 35 % |  |
|  | Progressive disease | 27 | 40 % | 30 | 41 % | 29 | 31 % | 86 | 36 % |  |
|  | Not available | 21 | - | 13 | - | 14 | - | 49 | - | - |
| Response third line | Partial/complete response | 4 | 15 % | 8 | 28 % | 14 | 26 % | 26 | 24 % | 0.784 |
|  | Stable disease | 8 | 31 % | 9 | 31 % | 14 | 26 % | 31 | 28 % |  |
|  | Progressive disease | 14 | 54 % | 12 | 41 % | 26 | 48 % | 52 | 48 % |  |
|  | Not available | 13 | - | 8 | - | 7 | - | 28 | - | - |
| *5 with unknown primary tumours excluded not presented separately, Adjuvant and neo-adjuvant, and Doublet+biologic and Triplet±biologic counted in the same category for Chi-square/Fisher´s exact test analysis, N/A=Not available | | | | | | | | | | |

**Table S5. Systemic therapy according to mutation status**

|  |  | *BRAF*-V600Emt | | RASmt | | RAS&*BRAF*wt | | Total* | | p-value |
| --- | --- | --- | --- | --- | --- | --- | --- | --- | --- | --- |
|  |  | 75 | 15 % | 264 | 52 % | 156 | 31 % | 508 | 100 % |  |
| Number of treatment | 1 | 40 | 53 % | 110 | 42 % | 61 | 39 % | 223 | 44 % | 0.010 |
| lines | 2 | 26 | 35 % | 80 | 30 % | 41 | 26 % | 148 | 29 % |  |
|  | 3+ | 9 | 12 % | 74 | 28 % | 54 | 35 % | 137 | 27 % |  |
| First line intent | Palliative | 62 | 83 % | 178 | 67 % | 98 | 63 % | 349 | 69 % | 0.002 |
|  | Conversion | 9 | 12 % | 28 | 11 % | 30 | 19 % | 67 | 13 % |  |
|  | Neo-adjuvant | 3 | 4 % | 40 | 15 % | 18 | 12 % | 62 | 12 % |  |
|  | Adjuvant | 1 | 1 % | 18 | 7 % | 10 | 6 % | 30 | 6 % |  |
| First line treatment | Single | 13 | 17 % | 56 | 21 % | 25 | 16 % | 100 | 20 % | 0.001 |
|  | Single+biologic | 6 | 8 % | 17 | 6 % | 16 | 10 % | 42 | 8 % |  |
|  | Doublet | 41 | 55 % | 137 | 52 % | 57 | 37 % | 239 | 47 % |  |
|  | Doublet+biologic | 10 | 13 % | 46 | 17 % | 46 | 29 % | 102 | 20 % |  |
|  | Triplet±biologic | 3 | 4 % | 7 | 3 % | 11 | 7 % | 21 | 4 % |  |
|  | Other | 2 | 3 % | 1 | 0 % | 1 | 1 % | 4 | 1 % | - |
| Second line treatment | Single | 2 | 6 % | 15 | 10 % | 6 | 6 % | 23 | 8 % | 0.002 |
|  | Single+biologic | 1 | 3 % | 27 | 18 % | 32 | 34 % | 60 | 21 % |  |
|  | Doublet | 18 | 51 % | 65 | 42 % | 25 | 26 % | 109 | 38 % |  |
|  | Doublet+biologic | 9 | 26 % | 42 | 27 % | 30 | 32 % | 81 | 28 % |  |
|  | Triplet±biologic | 0 | 0 % | 0 | 0 % | 1 | 1 % | 1 | 0 % |  |
|  | Other | 5 | 14 % | 5 | 3 % | 1 | 1 % | 11 | 4 % | - |
| Third line treatment | Single | 0 | 0 % | 8 | 11 % | 5 | 9 % | 13 | 9 % | N/A |
|  | Single+biologic | 2 | 22 % | 10 | 13 % | 20 | 37 % | 32 | 23 % |  |
|  | Doublet | 1 | 11 % | 23 | 31 % | 8 | 15 % | 32 | 23 % |  |
|  | Doublet+biologic | 2 | 22 % | 12 | 16 % | 15 | 28 % | 29 | 21 % |  |
|  | Other | 4 | 44 % | 22 | 29 % | 6 | 11 % | 32 | 23 % | - |
| Fourth line treatment | Single | 0 | 0 % | 0 | 0 % | 1 | 6 % | 1 | 2 % | N/A |
|  | Single+biologic | 0 | 0 % | 1 | 4 % | 5 | 29 % | 6 | 13 % |  |
|  | Doublet | 0 | 0 % | 5 | 21 % | 1 | 6 % | 6 | 13 % |  |
|  | Doublet+biologic | 3 | 50 % | 7 | 29 % | 1 | 6 % | 11 | 23 % |  |
|  | Other | 3 | 50 % | 11 | 46 % | 9 | 53 % | 23 | 49 % | - |
| Response first line | Partial/complete response | 13 | 25 % | 91 | 46 % | 70 | 56 % | 177 | 46 % | <0.001 |
|  | Stable disease | 7 | 13 % | 62 | 31 % | 31 | 25 % | 103 | 27 % |  |
|  | Progressive disease | 32 | 62 % | 47 | 24 % | 23 | 19 % | 107 | 28 % |  |
|  | Not available | 23 | - | 64 | - | 32 | - | 121 | - | - |
| Response second line | Partial/complete response | 8 | 30 % | 29 | 24 % | 30 | 35 % | 68 | 29 % | 0.271 |
|  | Stable disease | 7 | 26 % | 45 | 37 % | 30 | 35 % | 82 | 35 % |  |
|  | Progressive disease | 12 | 44 % | 49 | 40 % | 25 | 29 % | 86 | 36 % |  |
|  | Not available | 8 | - | 31 | - | 10 | - | 49 | - | - |
| Response third line | Partial/complete response | 1 | 17 % | 13 | 22 % | 12 | 28 % | 26 | 24 % | N/A |
|  | Stable disease | 0 | 0 % | 17 | 28 % | 14 | 33 % | 31 | 28 % |  |
|  | Progressive disease | 5 | 83 % | 30 | 50 % | 17 | 40 % | 52 | 48 % |  |
|  | Not available | 3 | - | 14 | - | 11 | - | 28 | - | - |
| *57 non-analysed tumours not presented separately, Adjuvant and Neo-adjuvant, and Doublet+biologic and Triplet±biologic counted in the same category for Chi-square/Fisher´s exact test analysis, N/A=Not available | | | | | | | | | | |

**Table S6. Treatment according to primary tumour location in the RAS&*BRAF* wildtype subgroup**

|  |  | Right colon | | Left colon | | Rectum | | Total | | p-value |
| --- | --- | --- | --- | --- | --- | --- | --- | --- | --- | --- |
|  |  | 57 | 100 % | 86 | 100 % | 77 | 100 % | 220 | 100 % |  |
| Type of treatment | Metastasectomy | 17 | 30 % | 30 | 35 % | 32 | 42 % | 79 | 36 % | 0.660 |
|  | Systemic therapy only | 25 | 44 % | 37 | 43 % | 31 | 40 % | 93 | 42 % |  |
|  | Best supportive care | 15 | 26 % | 19 | 22 % | 14 | 18 % | 48 | 22 % |  |
| Total chemotherapy |  | 40 | 100 % | 62 | 100 % | 54 | 100 % | 156 | 100 % | - |
| Number of treatment | 1 | 17 | 43 % | 19 | 31 % | 25 | 46 % | 61 | 39 % | 0.245 |
| lines | 2 | 9 | 23 % | 22 | 35 % | 10 | 19 % | 41 | 26 % |  |
|  | 3+ | 14 | 35 % | 21 | 34 % | 19 | 35 % | 54 | 35 % |  |
| First line intent | Palliative | 28 | 70 % | 37 | 60 % | 33 | 61 % | 98 | 63 % | 0.488 |
|  | Conversion | 4 | 10 % | 13 | 21 % | 13 | 24 % | 30 | 19 % |  |
|  | Neo-adjuvant | 4 | 10 % | 9 | 15 % | 5 | 9 % | 18 | 12 % |  |
|  | Adjuvant | 4 | 10 % | 3 | 5 % | 3 | 6 % | 10 | 6 % |  |
| First line treatment | Single | 8 | 20 % | 11 | 18 % | 6 | 11 % | 25 | 16 % | 0.247 |
|  | Single+biologic | 6 | 15 % | 5 | 8 % | 5 | 9 % | 16 | 10 % |  |
|  | Doublet | 15 | 38 % | 26 | 42 % | 16 | 30 % | 57 | 37 % |  |
|  | Doublet+biologic | 11 | 28 % | 14 | 23 % | 21 | 39 % | 46 | 29 % |  |
|  | Triplet±biologic | 0 | 0 % | 5 | 8 % | 6 | 11 % | 11 | 7 % |  |
|  | Other | 0 | 0 % | 1 | 2 % | 0 | 0 % | 1 | 1 % |  |
| Second line treatment | Single | 1 | 4 % | 5 | 12 % | 0 | 0 % | 6 | 6 % | 0.072 |
|  | Single+biologic | 8 | 35 % | 17 | 40 % | 7 | 24 % | 32 | 34 % |  |
|  | Doublet | 9 | 39 % | 9 | 21 % | 7 | 24 % | 25 | 26 % |  |
|  | Doublet+biologic | 4 | 17 % | 11 | 26 % | 15 | 52 % | 30 | 32 % |  |
|  | Triplet±biologic | 0 | 0 % | 1 | 2 % | 0 | 0 % | 1 | 1 % |  |
|  | Other | 1 | 4 % | 0 | 0 % | 0 | 0 % | 1 | 1 % |  |
| Third line treatment | Single | 1 | 7 % | 2 | 10 % | 2 | 11 % | 5 | 9 % | 0.877 |
|  | Single+biologic | 5 | 36 % | 8 | 38 % | 7 | 37 % | 20 | 37 % |  |
|  | Doublet | 1 | 7 % | 5 | 24 % | 2 | 11 % | 8 | 15 % |  |
|  | Doublet+biologic | 5 | 36 % | 5 | 24 % | 5 | 26 % | 15 | 28 % |  |
|  | Other | 2 | 14 % | 1 | 5 % | 3 | 16 % | 6 | 11 % |  |
| Fourth line treatment | Single | 0 | 0 % | 1 | 13 % | 0 | 0 % | 1 | 6 % | - |
|  | Single+biologic | 2 | 67 % | 2 | 25 % | 1 | 17 % | 5 | 29 % |  |
|  | Doublet | 0 | 0 % | 0 | 0 % | 1 | 17 % | 1 | 6 % |  |
|  | Doublet+biologic | 0 | 0 % | 0 | 0 % | 1 | 17 % | 1 | 6 % |  |
|  | Other | 1 | 33 % | 5 | 63 % | 3 | 50 % | 9 | 53 % |  |
| Response first line | Partial/complete response | 11 | 42 % | 26 | 49 % | 33 | 73 % | 70 | 56 % | 0.025 |
|  | Stable disease | 11 | 42 % | 14 | 26 % | 6 | 13 % | 31 | 25 % |  |
|  | Progressive disease | 4 | 15 % | 13 | 25 % | 6 | 13 % | 23 | 19 % |  |
|  | Not available | 31 | - | 33 | - | 32 | - | 96 | - | - |
| Response second line | Partial/complete response | 3 | 14 % | 16 | 43 % | 11 | 41 % | 30 | 35 % | 0.002 |
|  | Stable disease | 10 | 48 % | 6 | 16 % | 14 | 52 % | 30 | 35 % |  |
|  | Progressive disease | 8 | 38 % | 15 | 41 % | 2 | 7 % | 25 | 29 % |  |
|  | Not available | 2 | - | 6 | - | 2 | - | 10 | - | - |
| Response third line | Partial/complete response | 1 | 11 % | 4 | 25 % | 7 | 39 % | 12 | 28 % | 0.295 |
|  | Stable disease | 5 | 56 % | 6 | 38 % | 3 | 17 % | 14 | 33 % |  |
|  | Progressive disease | 3 | 33 % | 6 | 38 % | 8 | 44 % | 17 | 40 % |  |
|  | Not available | 5 | - | 5 | - | 1 | - | 11 | - | - |
| Adjuvant and Neo-adjuvant, and Doublet+biologic and Triplet±biologic counted in the same category for Chi-square/Fisher´s exact test analysis, N/A=Not available | | | | | | | | | | |

**Table S7. Systemic therapy according to mismatch repair status**

|  |  | pMMR | | dMMR | | Not tested | | Total | | p-value* |
| --- | --- | --- | --- | --- | --- | --- | --- | --- | --- | --- |
|  |  | 421 | 83 % | 28 | 6 % | 59 | 12 % | 508 | 100 % |  |
| Number of treatment | 1 | 176 | 42 % | 16 | 57 % | 31 | 53 % | 223 | 44 % | 0.283 |
| lines | 2 | 124 | 29 % | 6 | 21 % | 18 | 31 % | 148 | 29 % |  |
|  | 3+ | 121 | 29 % | 6 | 21 % | 10 | 17 % | 137 | 27 % |  |
| First line intent | Palliative | 279 | 66 % | 21 | 75 % | 49 | 83 % | 349 | 69 % | 0.799 |
|  | Conversion | 58 | 14 % | 3 | 11 % | 6 | 10 % | 67 | 13 % |  |
|  | Neo-adjuvant | 56 | 13 % | 3 | 11 % | 3 | 5 % | 62 | 12 % |  |
|  | Adjuvant | 28 | 7 % | 1 | 4 % | 1 | 2 % | 30 | 6 % |  |
| First line treatment | Single | 81 | 19 % | 4 | 14 % | 15 | 25 % | 100 | 20 % | 0.725 |
|  | Single+biologic | 31 | 7 % | 3 | 11 % | 8 | 14 % | 42 | 8 % |  |
|  | Doublet | 205 | 49 % | 11 | 39 % | 23 | 39 % | 239 | 47 % |  |
|  | Doublet+biologic | 84 | 20 % | 6 | 21 % | 12 | 20 % | 102 | 20 % |  |
|  | Triplet±biologic | 19 | 5 % | 1 | 4 % | 1 | 2 % | 21 | 4 % |  |
|  | Other | 1 | 0 % | 3 | 11 % | 0 | 0 % | 4 | 1 % | - |
| Second line treatment | Single | 19 | 8 % | 0 | 0 % | 4 | 14 % | 23 | 8 % | 0.707 |
|  | Single+biologic | 52 | 21 % | 3 | 25 % | 5 | 18 % | 60 | 21 % |  |
|  | Doublet | 97 | 40 % | 4 | 33 % | 8 | 29 % | 109 | 38 % |  |
|  | Doublet+biologic | 68 | 28 % | 2 | 17 % | 11 | 39 % | 81 | 28 % |  |
|  | Triplet±biologic | 1 | 0 % | 0 | 0 % | 0 | 0 % | 1 | 0 % |  |
|  | Other | 8 | 3 % | 3 | 25 % | 0 | 0 % | 11 | 4 % | - |
| Third line treatment | Single | 12 | 10 % | 0 | 0 % | 1 | 10 % | 13 | 9 % | 0.933 |
|  | Single+biologic | 30 | 25 % | 1 | 17 % | 1 | 10 % | 32 | 23 % |  |
|  | Doublet | 29 | 24 % | 1 | 17 % | 2 | 20 % | 32 | 23 % |  |
|  | Doublet+biologic | 26 | 21 % | 1 | 17 % | 2 | 20 % | 29 | 21 % |  |
|  | Other | 25 | 20 % | 3 | 50 % | 4 | 40 % | 32 | 23 % | - |
| Fourth line treatment | Single | 1 | 3 % | 0 | 0 % | 0 | 0 % | 1 | 2 % | N/A |
|  | Single+biologic | 5 | 13 % | 0 | 0 % | 1 | 50 % | 6 | 13 % |  |
|  | Doublet | 5 | 13 % | 0 | 0 % | 1 | 50 % | 6 | 13 % |  |
|  | Doublet+biologic | 9 | 23 % | 2 | 40 % | 0 | 0 % | 11 | 23 % |  |
|  | Other | 20 | 50 % | 3 | 60 % | 0 | 0 % | 23 | 49 % |  |
| Response first line | Partial/complete response | 156 | 48 % | 6 | 33 % | 15 | 32 % | 177 | 46 % | 0.105 |
|  | Stable disease | 85 | 26 % | 3 | 17 % | 15 | 32 % | 103 | 27 % |  |
|  | Progressive disease | 81 | 25 % | 9 | 50 % | 17 | 36 % | 107 | 28 % |  |
|  | Not available | 99 | - | 10 | - | 12 | - | 121 | - | - |
| Response second line | Partial/complete response | 66 | 33 % | 0 | 0 % | 2 | 8 % | 68 | 29 % | N/A |
|  | Stable disease | 68 | 34 % | 4 | 44 % | 10 | 40 % | 82 | 35 % |  |
|  | Progressive disease | 68 | 34 % | 5 | 56 % | 13 | 52 % | 86 | 36 % |  |
|  | Not available | 43 | - | 3 | - | 3 | - | 49 | - | - |
| Response third line | Partial/complete response | 25 | 26 % | 0 | 0 % | 1 | 11 % | 26 | 24 % | N/A |
|  | Stable disease | 27 | 28 % | 1 | 25 % | 3 | 33 % | 31 | 28 % |  |
|  | Progressive disease | 44 | 46 % | 3 | 75 % | 5 | 56 % | 52 | 48 % |  |
|  | Not available | 25 | - | 2 | - | 1 | - | 28 | - | - |
| *p-value between pMMR and dMMR, Adjuvant and Neo-adjuvant, and Doublet+biologic and Triplet±biologic counted in the same category for Chi-square analysis, dMMR=deficient mismatch repair, N/A=Not available, pMMR=proficient mismatch repair | | | | | | | | | | |

**Table S8 (A-B). Fit for intensive therapy based on baseline age and ECOG performance status only (A) and eligible for intensive therapy based on actual treatment allocation (B) divided by primary tumour location, mutational status, and mismatch repair status**

**A**

|  |  | Total | | Fit for intensive therapy* | | Not fit for intensive therapy | | | | | |
| --- | --- | --- | --- | --- | --- | --- | --- | --- | --- | --- | --- |
|  |  |  |  |  |  | Total | | Reasons for unfit | | | |
|  |  |  |  |  |  |  | | Age >75 years | | ECOG PS 2-4 | |
|  |  | 759 | 100 % | 335 | 44 % | 424 | 56 % | 296 | 39 % | 266 | 35 % |
| **Right colon** | RAS&*BRAF*wt | 44 | 100 % | 23 | 52 % | 21 | 48 % | 17 | 39 % | 11 | 25 % |
|  | RASmt | 100 | 100 % | 45 | 45 % | 55 | 55 % | 41 | 41 % | 30 | 30 % |
|  | *BRAF*-V600Emt | 49 | 100 % | 14 | ***29 %*** | 35 | 71 % | 21 | 43 % | 25 | ***51 %*** |
| **Left colon** | RAS&*BRAF*wt | 68 | 100 % | 36 | 53 % | 32 | 47 % | 21 | 31 % | 22 | 32 % |
|  | RASmt | 73 | 100 % | 37 | 51 % | 36 | 49 % | 25 | 34 % | 24 | 33 % |
|  | *BRAF*-V600Emt | 18 | 100 % | 6 | 33 % | 12 | 67 % | 6 | 33 % | 8 | 44 % |
| **Rectum** | RAS&*BRAF*wt | 67 | 100 % | 36 | 54 % | 31 | 46 % | 24 | 36 % | 14 | ***21 %*** |
|  | RASmt | 145 | 100 % | 86 | ***59 %*** | 59 | 41 % | 48 | 33 % | 34 | ***23 %*** |
|  | *BRAF*-V600Emt | 11 | 100 % | 6 | 55 % | 5 | 45 % | 2 | 18 % | 4 | 36 % |
| **Mismatch repair** | dMMR | 58 | 100 % | 18 | ***31 %*** | 40 | 69 % | 27 | 47 % | 28 | ***48 %*** |
| **Not tested** | Mutation or MMR | 126 | 100 % | 28 | ***22 %*** | 98 | ***78 %*** | 64 | ***51 %*** | 66 | ***52 %*** |
| BSC=best supportive care; dMMR=deficient mismatch repair, ECOG PS=Eastern Cooperative Oncology Group performance status. Statistically significant differences in bold and italic. *Fit for doublet/triplet chemotherapy+biologic or immune checkpoint inhibitors | | | | | | | | | | | |

**B**

|  |  | Total | | Eligible for intensive therapy* | | Not eligible for intense therapy | | | | | | | | | |
| --- | --- | --- | --- | --- | --- | --- | --- | --- | --- | --- | --- | --- | --- | --- | --- |
|  |  |  |  |  |  | Total | | Reasons for not eligible | | | | | | | |
|  |  |  |  |  |  |  |  | Unfit | | BSC only | | Curative metastasec-  tomy only£ | | One line & one drug§ | |
|  |  | 759 | 100 % | 272 | 36 % | 487 | 64 % | 424 | 56 % | 214 | 28 % | 84 | 11 % | 55 | 7 % |
| **Right colon** | RAS&*BRAF*wt | 44 | 100 % | 18 | 41 % | 26 | 59 % | 21 | 48 % | 9 | 20 % | 4 | 9 % | 3 | 7 % |
|  | RASmt | 100 | 100 % | 35 | 35 % | 65 | 65 % | 55 | 55 % | 27 | 27 % | 12 | 12 % | 10 | 10 % |
|  | *BRAF*-V600Emt | 49 | 100 % | 13 | 27 % | 36 | 73 % | 35 | 71 % | 16 | 33 % | 5 | 10 % | 3 | 6 % |
| **Left colon** | RAS&*BRAF*wt | 68 | 100 % | 31 | 46 % | 37 | 54 % | 32 | 47 % | 12 | ***18 %*** | 9 | 13 % | 2 | 3 % |
|  | RASmt | 73 | 100 % | 29 | 40 % | 44 | 60 % | 36 | 49 % | 15 | 21 % | 12 | 16 % | 7 | 10 % |
|  | *BRAF*-V600Emt | 18 | 100 % | 5 | 28 % | 13 | 72 % | 12 | 67 % | 3 | 17 % | 1 | 6 % | 2 | 11 % |
| **Rectum** | RAS&*BRAF*wt | 67 | 100 % | 30 | 45 % | 37 | 55 % | 31 | 46 % | 10 | ***15 %*** | 12 | 18 % | 3 | 4 % |
|  | RASmt | 145 | 100 % | 71 | ***49 %*** | 74 | 51 % | 59 | 41 % | 30 | ***21 %*** | 18 | 12 % | 9 | 6 % |
|  | *BRAF*-V600Emt | 11 | 100 % | 6 | 55 % | 5 | 45 % | 5 | 45 % | 3 | 27 % | 0 | 0 % | 1 | 9 % |
| **Mismatch repair** | dMMR | 58 | 100 % | 12 | ***21 %*** | 46 | 79 % | 40 | ***69 %*** | 27 | ***47 %*** | 6 | 10 % | 4 | 7 % |
| **Not tested** | Mutation or MMR | 126 | 100 % | 22 | ***17 %*** | 104 | ***83 %*** | 98 | ***78 %*** | 62 | ***49 %*** | 5 | 4 % | 11 | 9 % |
| BSC=best supportive care; dMMR=deficient mismatch repair, ECOG PS=Eastern Cooperative Oncology Group performance status. Statistically significant differences in bold and italic. *Fit for doublet/triplet chemotherapy+biologic or immune checkpoint inhibitors, £Has received only surgery with or without perioperative/adjuvant fluoropyrimidine±oxaliplatin, 49/84 patients were fit for intensive therapy §Only 7/55 patients were in in the fit for intensive therapy group according to age and ECOG PS. | | | | | | | | | | | | | | | |

**Figure S1 (A-D). Overall survival according to presentation of metastases in all patients (A), among right colon (B), left colon (C), and rectal primary tumours (D)**

**
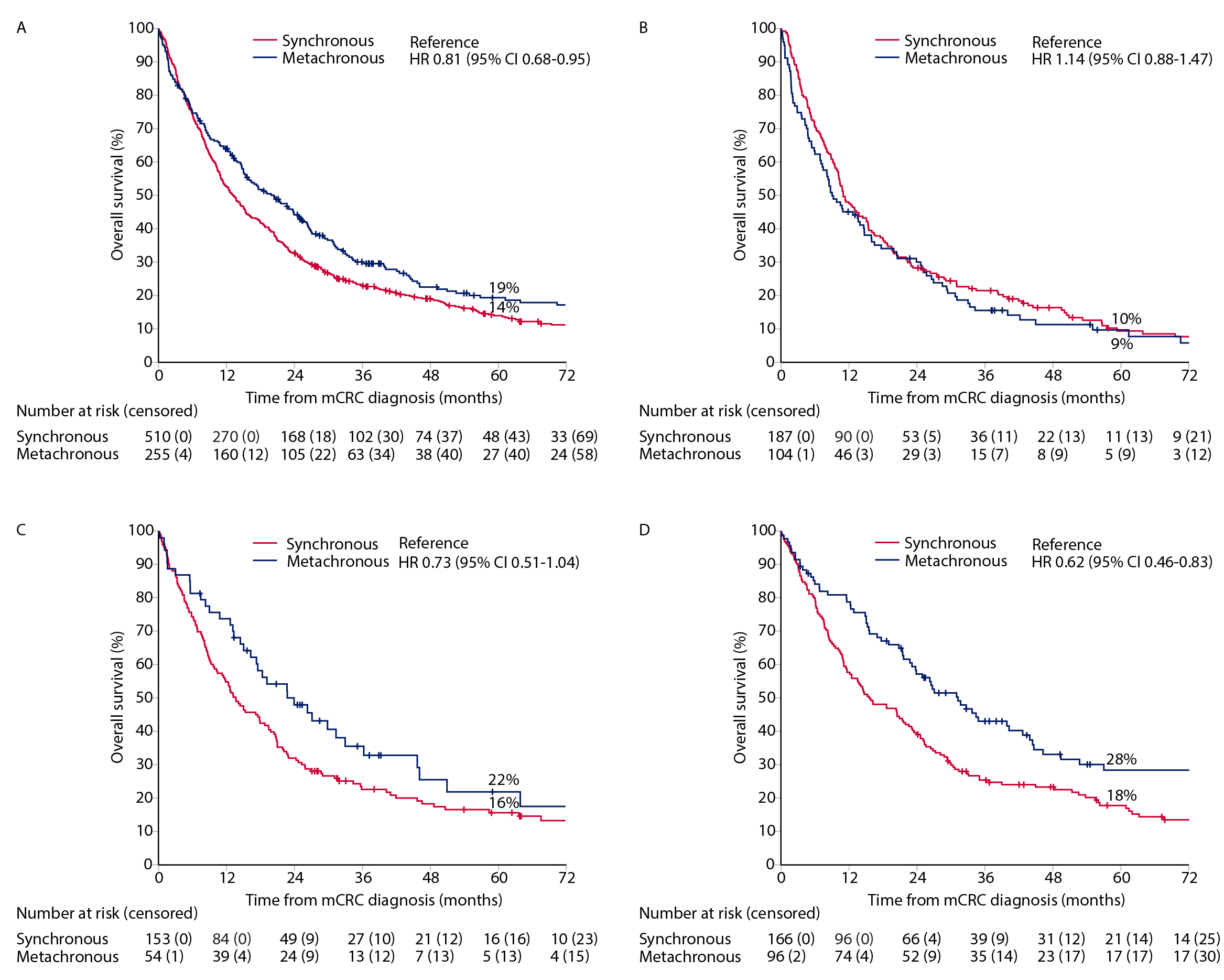
**

**Figure S2 (A-C). Overall survival according to treatment groups in all patients (A), in a conditional landmark analysis at 4 months (B), and a conditional landmark analysis at 3 weeks (C)**

**
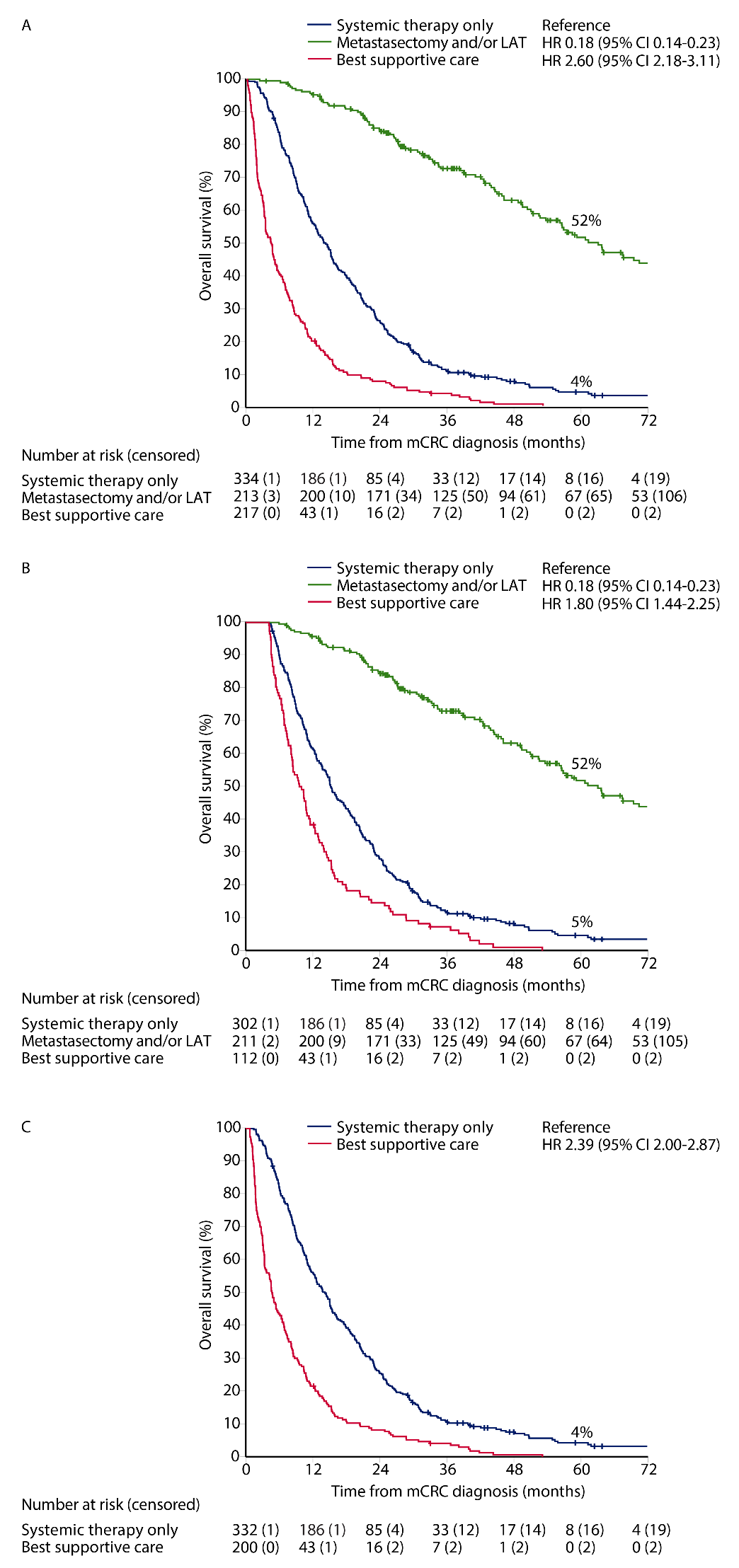
**

**Figure S3. Overall survival according to primary tumour location in a conditional landmark analysis at 4 months in the subgroup treated with metastasectomy**

**
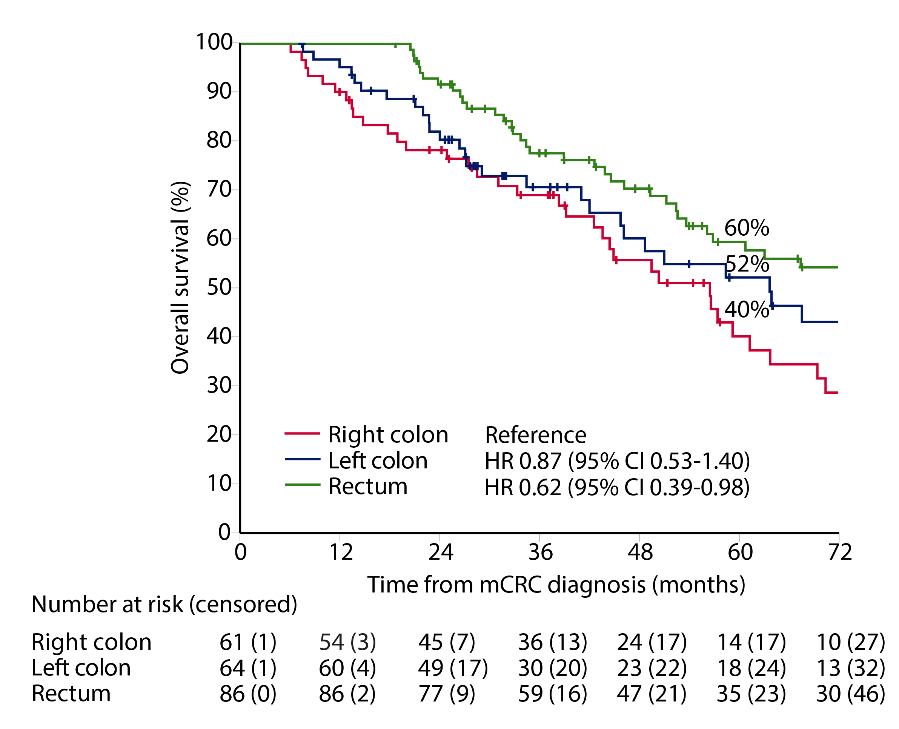
**

**Figure S4. Overall survival according to *PIK3CA* mutation status**


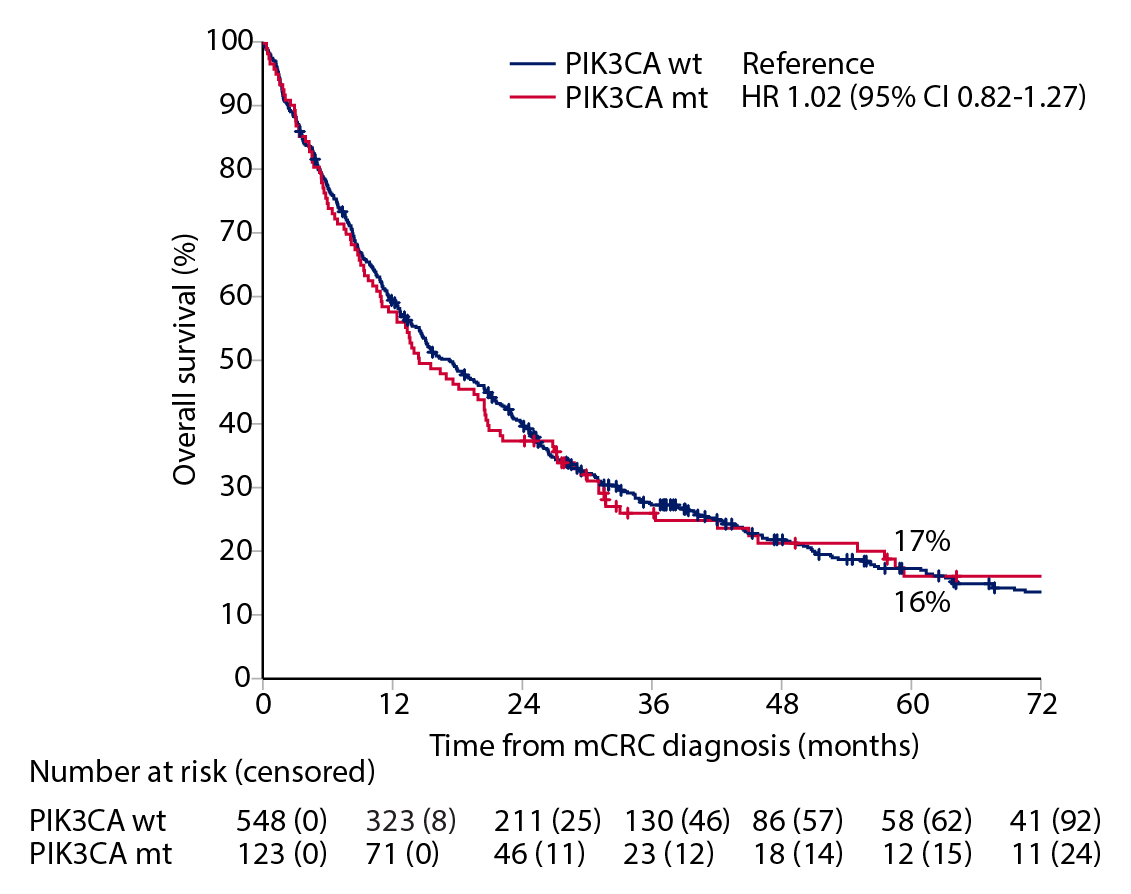


**Figure S5 (A-C). Overall survival according to primary tumour location in the RAS&*BRAF*wt and proficient mismatch repair subgroup separately for patients treated with systemic therapy only (A), metastasectomy (B), and best supportive care (C)**


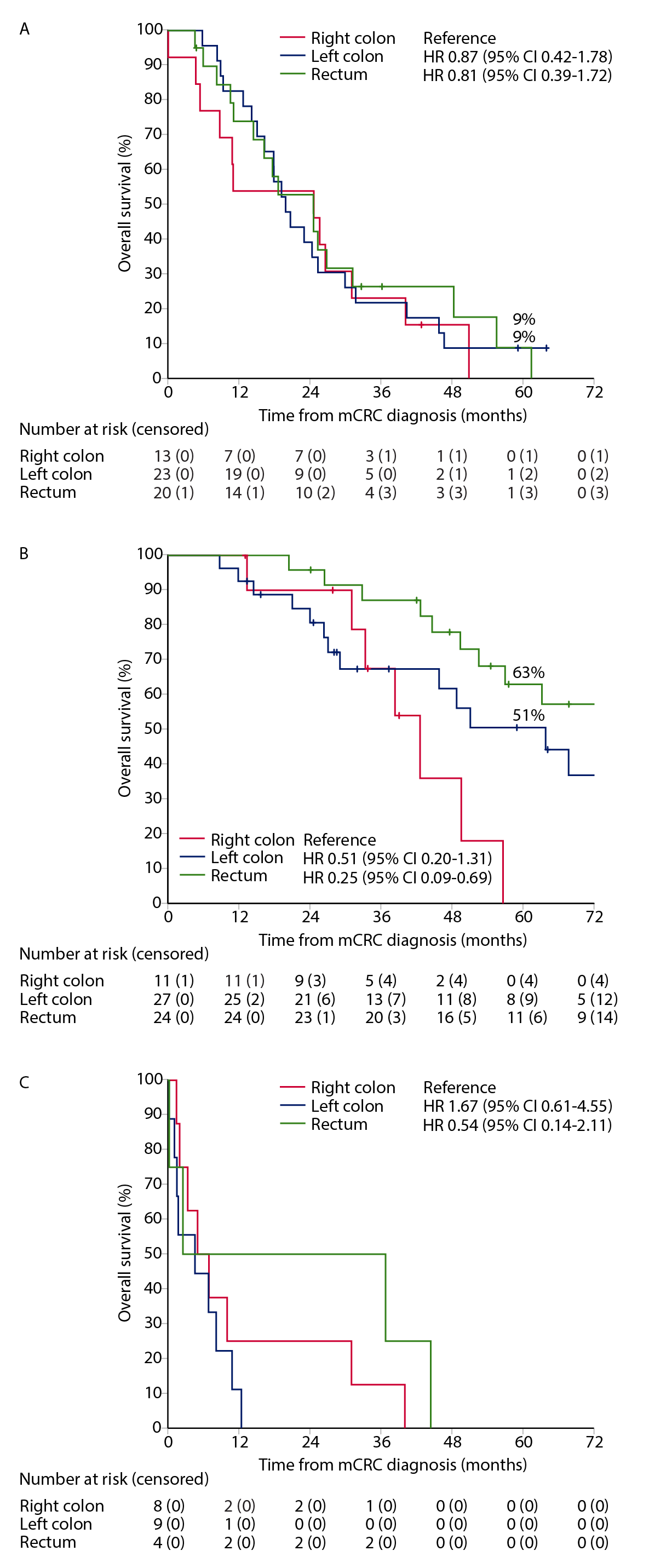


**Figure S6 (A-D). Overall survival according to the colorectal cancer continuum in all patients (A) and for patients with RAS&*BRAF* wildtype (A), RAS mutated (B), and *BRAF*-V600E mutated (C) tumours**

**
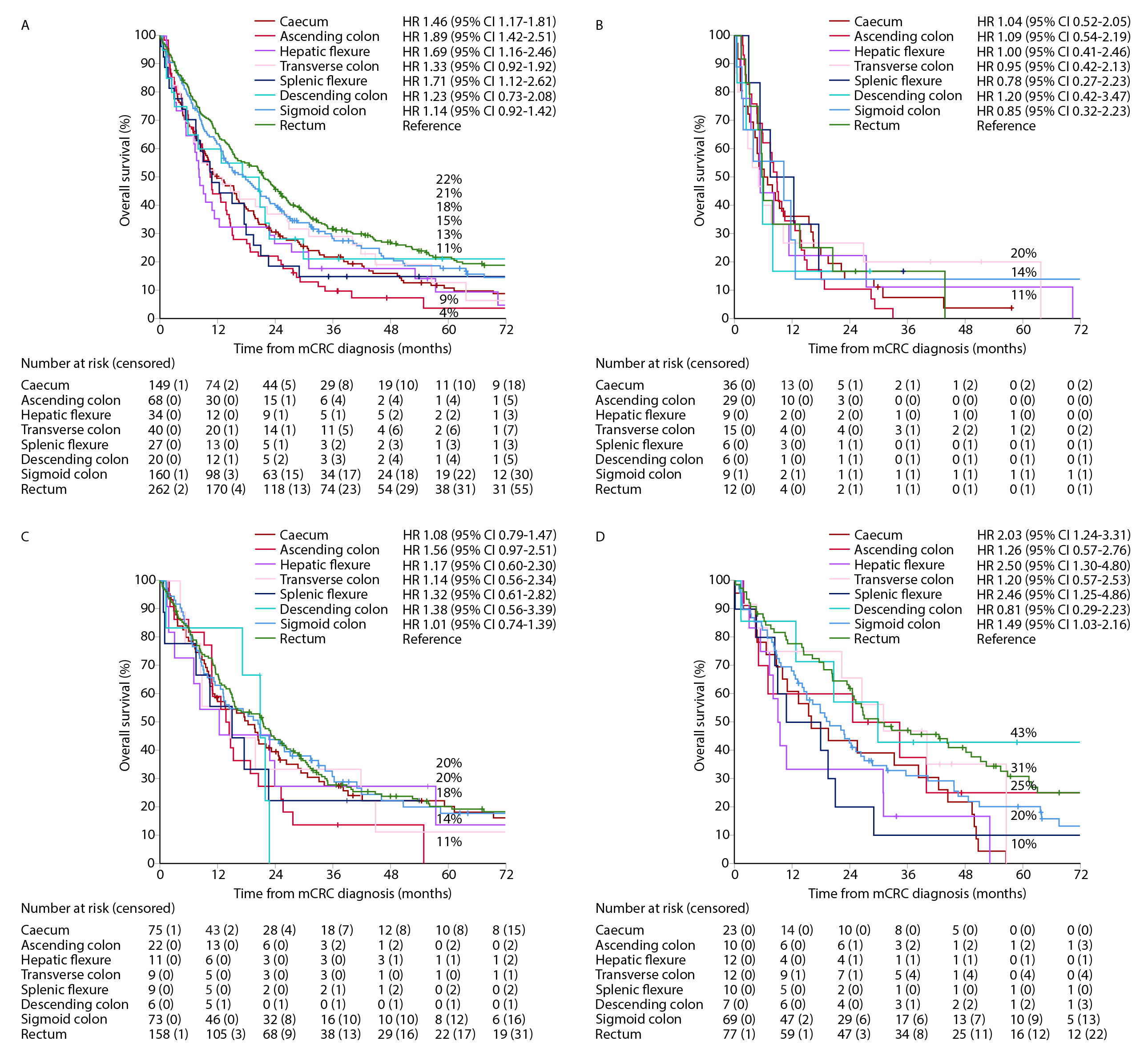
**

**Figure S7 (A-I). Overall survival according to mutation status among right colon primary tumours treated with metastasectomy (A) systemic therapy only (B), and best supportive care only (C), among left colon primary tumours treated with metastasectomy (D) systemic therapy only (E), and best supportive care only (F), and among rectal primary tumours treated with metastasectomy (G) systemic therapy only (H), and best supportive care only (I)**


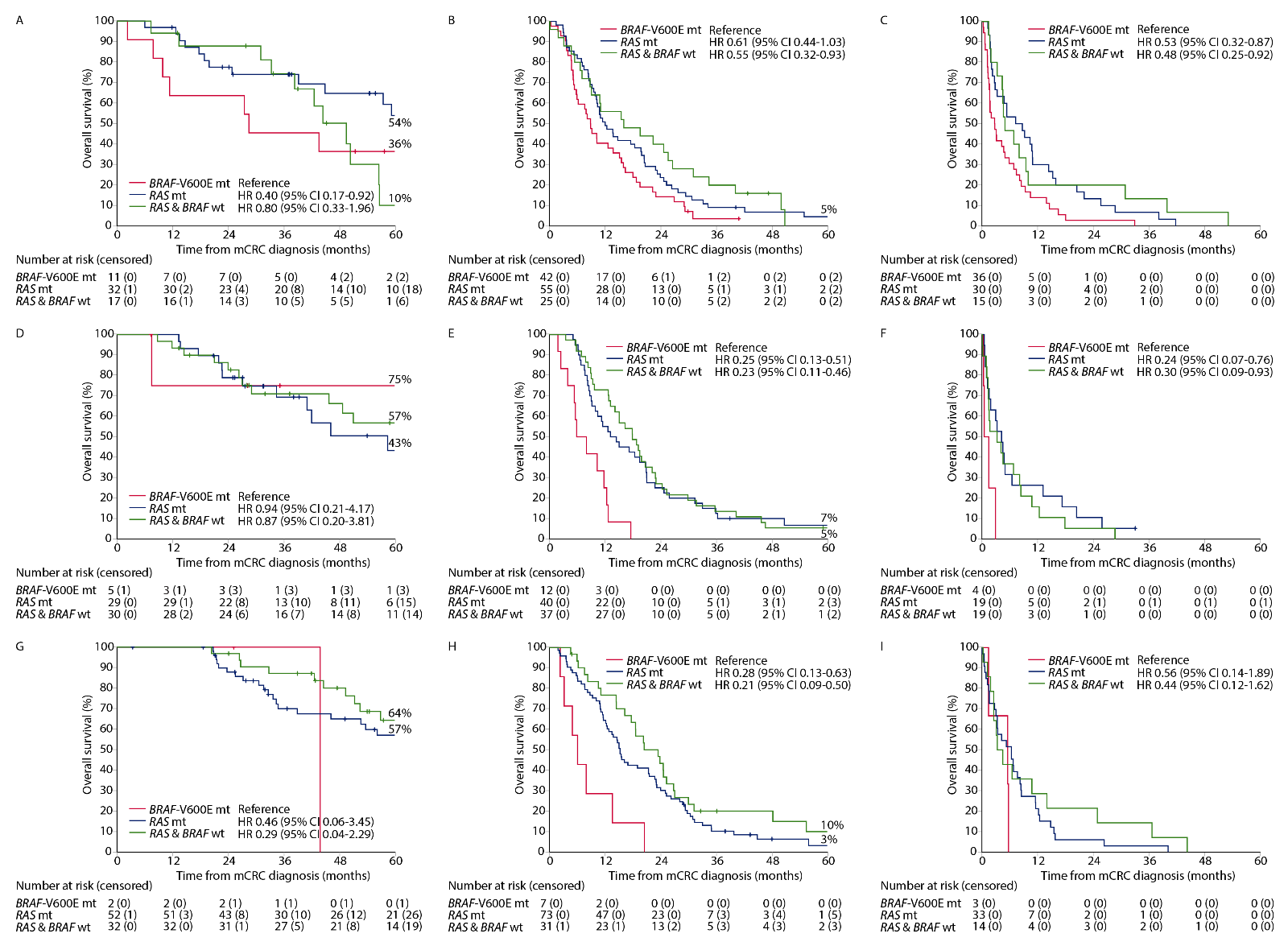


**Figure S8 (A-D). Overall survival according to mismatch repair status for all patients (A) and separately for patients treated with systemic therapy only (B), metastasectomy (C), and best supportive care only (D)**


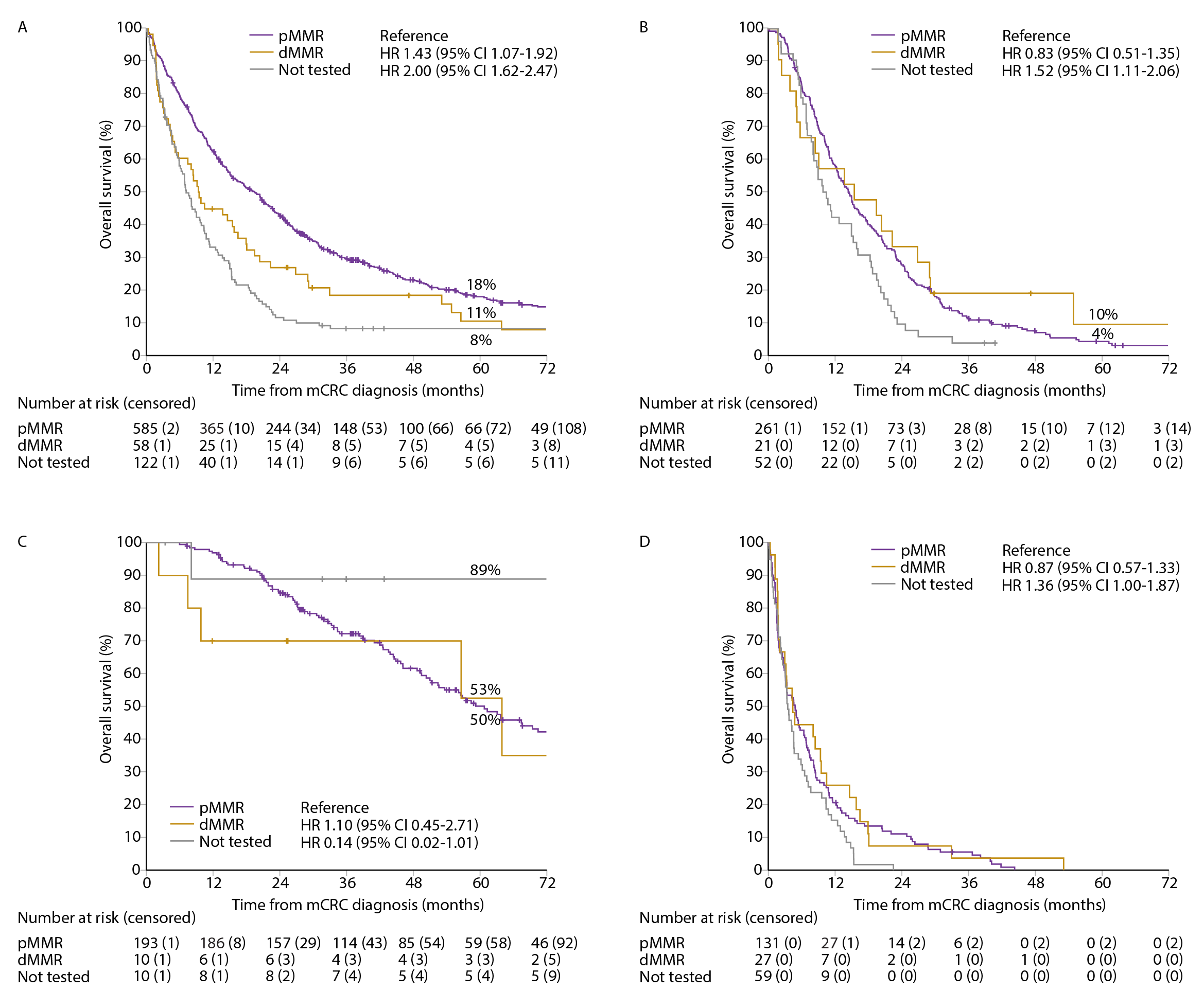


**Figure S9 (A-C). Overall survival according to mismatch repair status for patients with RAS&*BRAF* wildtype (A), RAS mutated (B), and *BRAF*-V600E mutated (C) tumours**

**
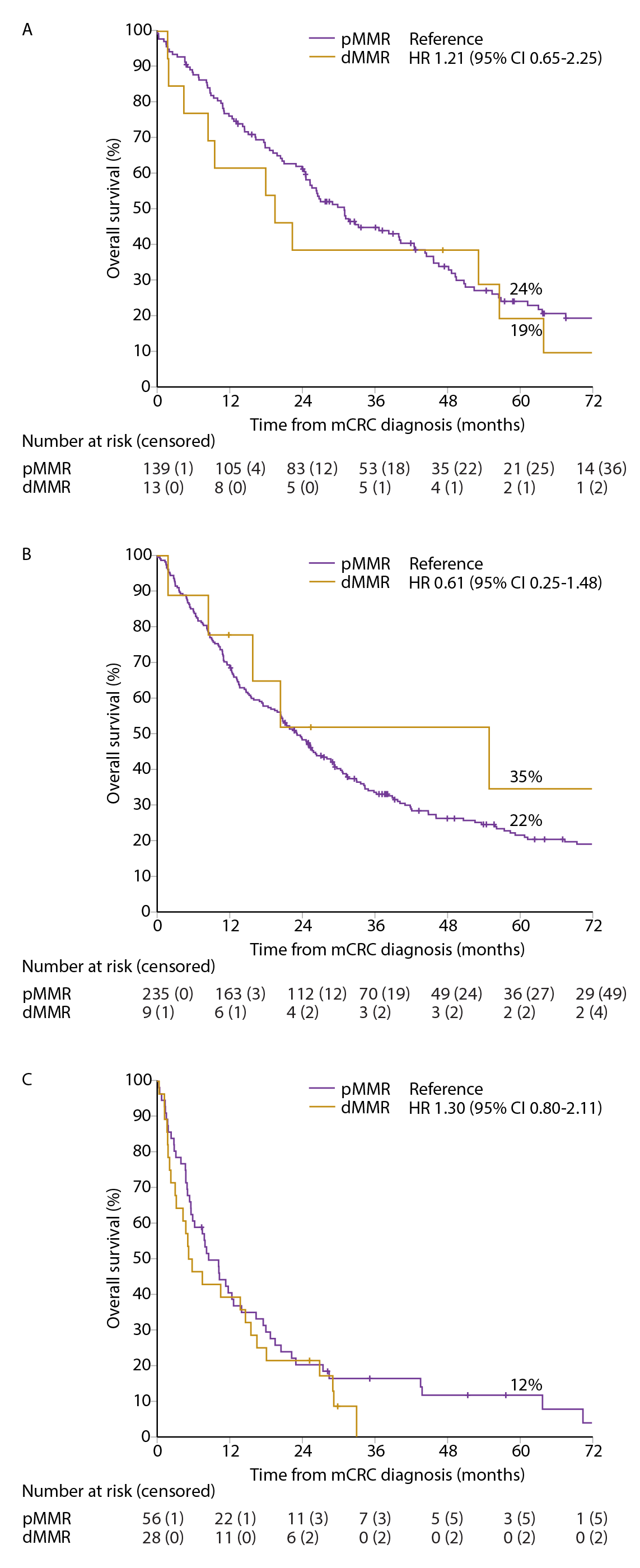
**
